# Complete Genome Sequence of 3-Chlorobenzoate-Degrading Bacterium *Cupriavidus necator* NH9 and Reclassification of the Strains of the Genera *Cupriavidus* and *Ralstonia* Based on Phylogenetic and Whole-Genome Sequence Analyses

**DOI:** 10.3389/fmicb.2019.00133

**Published:** 2019-02-12

**Authors:** Ryota Moriuchi, Hideo Dohra, Yu Kanesaki, Naoto Ogawa

**Affiliations:** ^1^Research Institute of Green Science and Technology, Shizuoka University, Shizuoka, Japan; ^2^The United Graduate School of Agricultural Science, Gifu University, Gifu, Japan; ^3^Graduate School of Agriculture, Shizuoka University, Shizuoka, Japan

**Keywords:** aromatic degradation, *Cupriavidus*, *Ralstonia*, reclassification, ANI (average nucleotide identity), TNA (tetra-nucleotide analysis)

## Abstract

*Cupriavidus necator* NH9, a 3-chlorobenzoate (3-CB)-degrading bacterium, was isolated from soil in Japan. In this study, the complete genome sequence of NH9 was obtained via PacBio long-read sequencing to better understand the genetic components contributing to the strain's ability to degrade aromatic compounds, including 3-CB. The genome of NH9 comprised two circular chromosomes (4.3 and 3.4 Mb) and two circular plasmids (427 and 77 kb) containing 7,290 coding sequences, 15 rRNA and 68 tRNA genes. Kyoto Encyclopedia of Genes and Genomes pathway analysis of the protein-coding sequences in NH9 revealed a capacity to completely degrade benzoate, 2-, 3-, or 4-hydroxybenzoate, 2,3-, 2,5-, or 3,4-dihydroxybenzoate, benzoylformate, and benzonitrile. To validate the identification of NH9, phylogenetic analyses (16S rRNA sequence-based tree and multilocus sequence analysis) and whole-genome sequence analyses (average nucleotide identity, percentage of conserved proteins, and tetra-nucleotide analyses) were performed, confirming that NH9 is a *C*. *necator* strain. Over the course of our investigation, we noticed inconsistencies in the classification of several strains that were supposed to belong to the two closely-related genera *Cupriavidus* and *Ralstonia*. As a result of whole-genome sequence analysis of 46 *Cupriavidus* strains and 104 *Ralstonia* strains, we propose that the taxonomic classification of 41 of the 150 strains should be changed. Our results provide a clear delineation of the two genera based on genome sequences, thus allowing taxonomic identification of strains belonging to these two genera.

## Introduction

The Gram-negative bacterial genera *Cupriavidus* and *Ralstonia* belong to the family *Burkholderiaceae* and the class β-proteobacteria. The two genera are closely related and have a complex taxonomic history, which was addressed by Yabuuchi et al. (1995) and Vandamme and Coenye (2004). The genus *Cupriavidus* was established in 2004 (Vandamme and Coenye, 2004), with members of this genus isolated from a variety of environments, including soil (Poehlein et al., 2011), ground water (Ray et al., 2015), activated sludge (Shafie et al., 2017), root nodules (Amadou et al., 2008), spacecraft-associated environments (Monsieurs et al., 2014), and human clinical specimens (Monsieurs et al., 2013). These divergent ecological niches explain the diversity of the genus, which currently comprises 17 species (http://www.bacterio.net/cupriavidus.html). To date, the genomes of a variety of *Cupriavidus* species have been sequenced, and show several common features. In particular, all *Cupriavidus* species examined have multi-replicon genomes, often including large plasmids, containing metal resistance genes and genes involved in the biodegradation of persistent aromatic compounds (Amadou et al., 2008; Janssen et al., 2010; Lykidis et al., 2010; Poehlein et al., 2011; Ray et al., 2015; Suenaga et al., 2015; Wang X. et al., 2015; Fang et al., 2016; Shafie et al., 2017). As halogenated or non-halogenated aromatic compounds are abundant in the environment as pollutants (e.g., chlorobenzenes and polychlorinated biphenyls, PCBs), understanding the degradation for these recalcitrant aromatics by microorganisms is of great interest for characterizing the behavior of soil-dwelling microorganisms and for the development of novel bioremediation processes (Reineke and Knackmuss, 1988).

*Cupriavidus necator* NH9 (formerly known as *Alcaligenes eutrophus* or *Ralstonia eutropha*) was isolated from a soil sample of the ground near a building of National Institute for Agro-Environmental Sciences (currently, Institute for Agro-Environmental Sciences, NARO) of Tsukuba city, Japan by using 3-chlorobenzoate (3-CB) as a sole source of carbon and energy (Ogawa and Miyashita, 1995). In strain NH9, 3-CB is thought to be first converted to 3- or 4-chlorocatechols by chromosomally-encoded enzymes. The resultant chlorocatechols are converted to β-ketoadipate, a central metabolite of soil bacteria, by the enzymes of the chlorocatechol *ortho*-cleavage pathway. These enzymes of strain NH9 are encoded by the *cbnABCD* genes, which are contained on plasmid pENH91 (Ogawa and Miyashita, 1995, 1999). Chlorocatechols are key intermediate metabolites in the aerobic microbial degradation pathways of various chlorinated aromatic compounds (Reineke, 1998). The genes for degradation of chlorocatechols are often encoded on large plasmids. For example, the *tfdCDEF, clcABDE*, and *tcbCDEF* genes encoding enzymes of chlorocatechol *ortho*-cleavage pathway are carried on the plasmids pJP4 of *Cupriavidus pinatubonensis* JMP134 (Don et al., 1985), pAC27 of *Pseudomonas putida* AC866 (Frantz and Chakrabarty, 1987; Kasberg et al., 1997) and pP51 of *Pseudomonas* sp. P51 (van der Meer et al., 1991), respectively. Accordingly, the genes encoding chlorocatechol *ortho*-cleavage pathway enzymes could spread beyond boundaries of bacterial species. In addition to their simple structures, the production of chlorocatechols as intermediates makes chlorobenzoates suitable model substrate compounds for the study of microbial degradation of chlorinated aromatics (Morimoto et al., 2005). Moreover, chlorobenzoates themselves are the intermediate products of the degradation of PCBs (Reineke and Knackmuss, 1988). In *Comamonas testosterone* BR60 (formerly *Alcaligenes* sp. BR60), 3-CB is known to be converted to 5-chloroprotocatechuate or protocatechuate by the products of the *cbaABC* genes and further metabolized via protocatechuate *meta*-ring fission pathway (Nakatsu et al., 1997). Several critical features of the ability of strain NH9 to degrade 3-CB have been characterized by analyses of the substrate specificity and application of chlorocatechol 1,2-dioxygenase (CbnA) (Liu et al., 2005; Ohmiya et al., 2009), and by biochemical and structural analyses of CbnR, a LysR-type transcriptional regulator controlling the expression of the *cbnABCD* genes (Moriuchi et al., 2017; Koentjoro et al., 2018). While these studies have been beneficial for gaining knowledge on both basic and applied aspects of the degradation ability of NH9 or its enzymes, genomic analysis of NH9 would provide further insights into the genes involved in the catabolism of aromatic compounds by this strain.

In the course of our analysis of the genetic characteristics of the strain NH9 to degrade aromatic compounds in comparison with related bacterial strains, we noticed inconsistency of phylogenetic identification of several strains of the genera *Cupriavidus* and *Ralstonia*, which is the genus most close to *Cupriavidus*. Thus, in order to precisely understand genetic characteristic of the strain NH9 among phylogenetically related bacteria, accurate taxonomic identification of NH9 is required.

The genus *Ralstonia* was first established by Yabuuchi et al. in 1995 to accommodate several misplaced species, including *A. eutrophus* (currently the genus *Cupriavidus*), *Burkholderia pickettii*, and *B. solanacearum* (Yabuuchi et al., 1995). As of May 2018, genome data of 104 strains that belong to four *Ralstonia* species have been deposited in the GenBank database. *Ralstonia solanacearum*, the most sequenced species, is an important phytopathogen that causes bacterial wilt in a variety of economically important crops (Hayward, 1991). *R*. *solanacearum* strains are divided into four phylotypes based on their geographic origins: Asia (phylotype I), America (IIA and IIB), Africa (III), and Indonesia-Japan (IV) (Castillo and Greenberg, 2007; Safni et al., 2014). The remaining three *Ralstonia* species, *Ralstonia pickettii, R*. *insidiosa*, and *R*. *mannitolilytica*, are commonly found in moist environments (e.g., water and soil) and are opportunistic human pathogens (Ryan and Adley, 2014). *R*. *pickettii* also has the capacity to degrade many toxic substances and, like *Cupriavidus* strains, is found in diverse habitats (Ryan et al., 2007).

Because of the decreasing cost of genome sequencing, a growing number of *Cupriavidus* and *Ralstonia* genomes are being sequenced and deposited in public databases. However, taxonomic problems have arisen at the species and genus levels because of the diversity and complex taxonomic history of these two closely related genera. While several studies aimed at inferring the phylogeny of *R*. *solanacearum* have been performed (Prior et al., 2016; Zhang and Qiu, 2016), the phylogenetic relationships between the genera *Cupriavidus* and *Ralstonia* have never been elucidated. In this study, the complete genome sequence of *C*. *necator* NH9 was revealed using PacBio long-reads-based sequencing, allowing us to infer its capacity for the degradation of aromatic compounds. The phylogenetic relationships between *Cupriavidus* and *Ralstonia* were also investigated based on whole-genome sequences. Overall, our findings provide a detailed and well-supported description of the phylogenetic relationships between these two genera.

## Materials and Methods

### Genomic DNA Extraction and Genome Sequencing and Assembly

*C*. *necator* NH9 was cultured in basal salts medium (Ogawa and Miyashita, 1995) containing 5 mM 3-CB as the sole source of carbon and energy at 30°C. NH9 genomic DNA was extracted using a DNeasy Blood and Tissue Kit (QIAGEN) and then used as template for whole-genome sequencing via the PacBio RSII system (Pacific Biosciences) by Macrogen Inc. (http://www.macrogen.com), with the resulting assembly confirmed using the MiSeq platform (Illumina) at the Instrumental Research Support Office, Research Institute of Green Science and Technology, Shizuoka University. PacBio RSII sequencing produced 181,370 raw reads, which were filtered using PreAssembler Filter v1 of the RS HGAP Assembly.3 Protocol in SMRT Analysis Software version 2.3.0 (Chin et al., 2013). A minimum polymerase read quality cut-off of 0.75 and a minimum subread length of 7.5 kb were used. We obtained a total of 86,406 filtered subreads, with an *N*_50_ read length of 12,367 bp and a max read length of 41,609 bp, resulting in 1,050,061,719 bp of sequence with ~127-fold coverage. These high quality subreads were then *de novo* assembled using HGAP.3 (Chin et al., 2013) with a minimum seed read length of 15 kb. The resulting four contigs were polished using AssemblyPolishing v1 Quiver (RS HGAP Assembly.3 Protocol) and Arrow (https://github.com/PacificBiosciences/GenomicConsensus), and then closed using Circlator version 1.1.1 (Hunt et al., 2015). To identify errors in the final PacBio assembled contigs, Illumina sequencing data were also collected. A paired-end library was constructed for MiSeq sequencing using a KAPA HyperPlus Kit (KAPA BIOSYSTEMS), resulting in 3,436,955 paired-end reads (2 × 301 bp). Low-quality reads (quality score, < Q15), adapter sequences, reads < 150 bp, and the terminal 301 bases were filtered using Trimmomatic version 0.33 (Bolger et al., 2014), yielding 2,305,131 paired reads corresponding to a coverage of ~138-fold. These high-quality short reads were aligned against the four polished circular contigs using BWA-MEM (Li, 2013) and manually checked using Integrative Genomics Viewer (Thorvaldsdottir et al., 2013). When an error was identified, the relevant position was manually curated. The final complete genome sequence of NH9 has been deposited in DDBJ/ENA/GenBank under accession numbers CP017757–CP017760.

### Genome Annotation

Four complete genome sequences of NH9 were annotated using the NCBI Prokaryotic Genome Automatic Annotation Pipeline (PGAAP) (Tatusova et al., 2016), the Microbial Genome Annotation Pipeline (MiGAP, http://www.migap.org), and Prokka software version 1.11 (Seemann, 2014). PGAAP annotation data was manually curated with respect to start codon position and missing genes by referencing it against the MiGAP and Prokka annotation data with the aid of GenomeMatcher (Ohtsubo et al., 2008), Geneious software version 11.0.4 (Kearse et al., 2012), BLASTP analysis (Altschul et al., 1997), and InterProScan (Jones et al., 2014). All putative proteins identified in the NH9 genome were functionally classified based on Clusters of Orthologous Groups (COG) analysis using RPS-BLAST (Altschul et al., 1997). BlastKOALA (Kanehisa et al., 2016a) was used for functional characterization of the NH9 complete genome to reconstruct aromatic compound degradation pathways using the Kyoto Encyclopedia of Genes and Genomes (KEGG) database (Kanehisa et al., 2016b).

### Genome Sequence Data Collection

All genome sequence data for the *Cupriavidus* and *Ralstonia* strains used in this study were obtained from the assembly summary report file (ftp://ftp.ncbi.nlm.nih.gov/genomes/ASSEMBLY_REPORTS/assembly_summary_refseq.txt). *R*. *pickettii* DTP0602 was also added manually. *R*. *solanacearum* BBAC-C1 was removed from all analyses because of low genome sequence coverage that adversely affected results. Complete or draft genome sequences, GenBank files, protein coding sequences, amino acid sequences, and RNA gene sequences for the 46 *Cupriavidus* and 104 *Ralstonia* named strains selected for analysis were downloaded from the NCBI FTP site (ftp://ftp.ncbi.nlm.nih.gov/genomes/all/) in May 2018.

### 16S rRNA and Multilocus Sequence Analysis

Phylogenetic analysis was performed using MEGA software version 7.0 (Kumar et al., 2016). For the 16S rRNA gene-based phylogenetic analysis, alignments were carried out using ClustalW and analysis was performed using the maximum likelihood method and the GTR + G substitution model. In addition to the genome-sequenced strains, the 16S rRNA genes of several *Cupriavidus* and *Ralstonia* type strains (*Cupriavidus basilensis, C. gilardii, C*. *oxalaticus, C*. *pauculus, C*. *pinatubonensis, R*. *insidiosa*, and *R*. *mannitolilytica*) were downloaded from the NCBI database. As only partial 16S rRNA gene sequences were available for *Cupriavidus metallidurans* NE12, *C*. *oxalaticus* NBRC 13593, *Cupriavidus taiwanensis* STM 6018, *C*. *taiwanensis* STM 6070, *Cupriavidus* sp. amp6, *Cupriavidus* sp. GA3-3, *Cupriavidus* sp. IDO, *Cupriavidus* sp. UYPR2.512, *R*. *solanacearum* P673, *R*. *solanacearum* Y45, and *R*. *solanacearum* Rs-10-244, these strains were not included in the analysis. Following alignment, all gaps were eliminated, resulting in a shared 1,386-bp sequence for the final analysis. The 16S rRNA gene sequence of *Paraburkholderia xenovorans* LB400 (Sawana et al., 2014) was used as the outgroup for the analysis. To evaluate the phylogenetic tree topology, a bootstrap analysis of 1,000 replicates was performed.

For the multilocus sequence analysis (MLSA), we screened for the presence of several single-copy housekeeping genes in the genomes of the *Cupriavidus* and *Ralstonia* strains. As a result, we found that the following four genes were present in all of the strains except *Cupriavidus* sp. SK-3, thus the four genes were used for MLSA: *atpD* (β-subunit of ATP synthase F_0_F_1_ gene), *leuS* (leucine-tRNA ligase gene), *rplB* (50S ribosomal protein L2 gene), and *gyrB* (β-subunit of DNA gyrase gene). Multiple alignments were performed with respect to each gene using ClustalW, and all positions containing gaps or missing data were excluded. All aligned genes were then concatenated in the following order: *atpD*-*leuS*-*rplB*-*gyrB*. The final lengths of each gene and the complete concatenated sequence were: *atpD*, 1,278 bp; *leuS*, 2,571 bp; *rplB*, 822 bp; *gyrB*, 1,662 bp; concatenated sequence, 6,333 bp. Maximum likelihood analysis using the GTR + G substitution model was performed with 1,000 bootstrap replicates. The corresponding *P*. *xenovorans* LB400 gene sequences were used as the outgroup for the analysis.

### Whole-Genome Comparisons

Average nucleotide identity (ANI) (Goris et al., 2007) and percentage of conserved proteins (POCP) (Qin et al., 2014) analyses were used to compare whole-genome sequences. The ANI value, resulting from the mean identity of BLASTN matches between the virtually-fragmented query and reference genomes, was calculated using ani.rb script from the enveomics collection (Rodriguez and Konstantinidis, 2016) with default settings. POCP was used to identify conserved proteins between a pair of genomes using BLASTP analysis and to provide accurate genus cut-off values. To calculate POCP values, a POCP script developed by Harris et al. (2017) was used with the following parameters: *E*-value < 1e^−5^, sequence identity ≥ 40%, and alignable region of the query protein sequences ≥ 50%. A dendrogram was constructed based on the Unweighted Pair Group Method with Arithmetic Mean clustering method with a distance of (1 – ANI) in R program version 3.4.4 (https://www.r-project.org/).

### Tetra-Nucleotide Analysis (TNA)

The tetra-nucleotide frequencies of all *Cupriavidus* and *Ralstonia* genome sequences were calculated using the compseq program from the EMBOSS package (http://emboss.sourceforge.net/apps/cvs/emboss/apps/compseq.html). Results of TNA were visualized by generating a three-dimensional plot of principal component analysis (PCA) in R package rgl. The frequencies of all 256 possible tetra-nucleotides were used as input for PCA.

## Results and Discussion

### General Properties and Structure of the NH9 Genome

**Figure 1:**
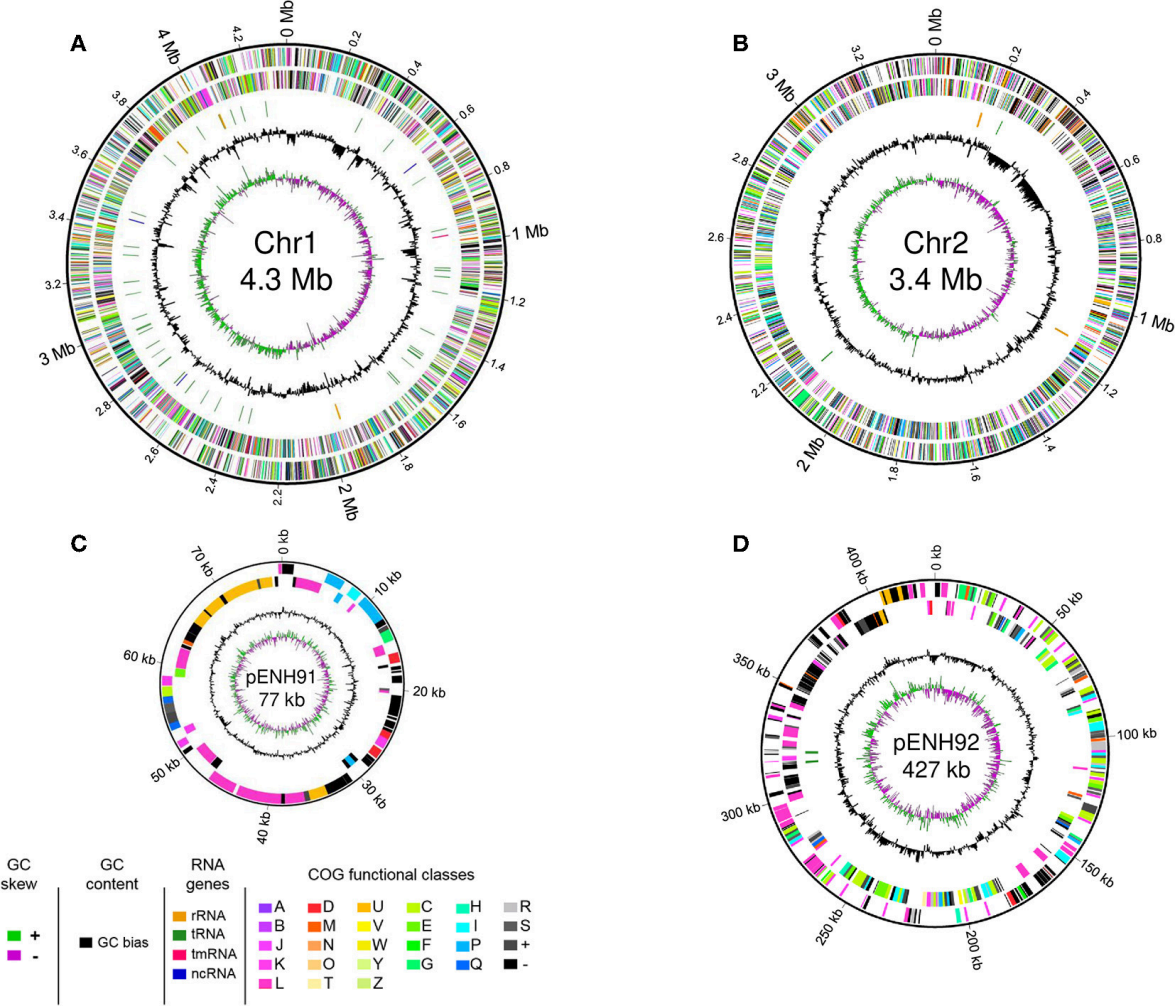
Schematic representation of the four replicons of NH9. Circular maps of chromosome 1 **(A)**, chromosome 2 **(B)**, pENH91 **(C)**, and pENH92 **(D)** are shown. The circles represent (from the inside): 1, GC skew; 2, GC content; 3, RNA genes (except for pENH91); 4, Clusters of Orthologous Groups (COG) assignments for coding sequences (CDSs) on the reverse strand; 5, COG assignments for CDSs on the forward strand; 6, scale in Mb or kb. Note that maps are not drawn to scale relative to the sizes of each replicon, very short RNA genes are enlarged to enhance visibility, and pENH91 does not contain any RNA genes.

Genome statistics are presented in Table 1 and a circular genome map is depicted in Figure 1. The genome of *C. necator* strain NH9 comprises two circular chromosomes (Chr), Chr1 (4,347,557 bp, 65.8% G+C) (Figure 1A) and Chr2 (3,395,604 bp, 65.5% G+C) (Figure 1B), along with two circular plasmids, pENH91 (77,172 bp, 64.2% G+C) (Figure 1C) and pENH92 (426,602 bp, 61.8% G+C) (Figure 1D). A total of 7,290 coding sequences (CDSs) were predicted by homology analysis. The NH9 genome contained 68 tRNA genes, two of which were located on pENH92. Chr1 and Chr2 contained three and two rRNA gene operons (5S, 16S, and 23S rRNA genes), respectively, while Chr1 also had one tmRNA and three ncRNAs. Although the G+C contents of all replicons were similar, those of the two plasmids, particularly pENH92, were lower than those of the two chromosomes (Table 1).

**Table 1:** General properties of the *C*. *necator* NH9 genome.

	**Chromosome 1**	**Chromosome 2**	**pENH91**	**pENH92**	**Genome**
Accession number	CP017757	CP017758	CP017760	CP017759	–
Sequence length (bp)	4,347,557	3,395,604	77,172	426,602	8,246,935
GC (%)	65.8	65.5	64.2	61.8	65.5
tRNA genes	57	9	0	2	68
tmRNA genes	1	0	0	0	1
ncRNA genes	3	0	0	0	3
rRNA gene operons	3	2	0	0	5
Coding (%)	87.3	86.7	92.2	85.2	87.0
Total number of CDSs	3,912	2,932	78	368	7,290
CDSs assigned to COG	3,166 (80.9%)	2,300 (78.4%)	46 (59.0%)	249 (67.7%)	5,761 (79.0%)
CDSs assigned to KEGG Ontology	2,828 (72.3%)	1,901 (64.8%)	52 (66.7%)	236 (64.1%)	5,017 (68.8%)

**Figure 2:**
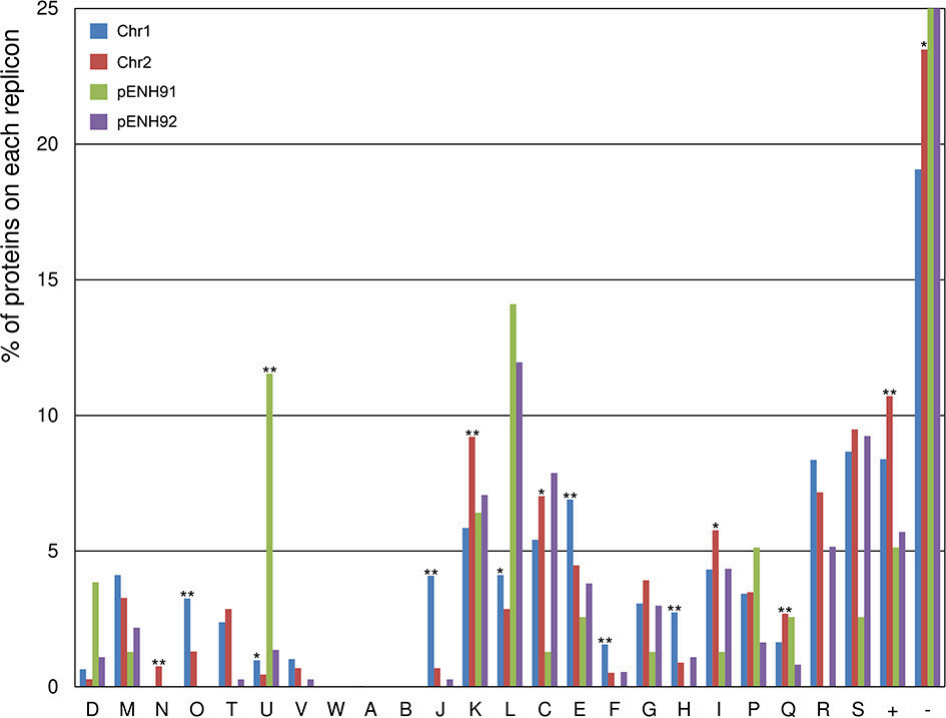
Functional classification of proteins encoded on each replicon of the NH9 genome based on Clusters of Orthologous Groups (COG) categories. COG categories are shown on the horizontal axis, with the percentage of proteins belonging to each category for each replicon plotted on the vertical axis (percentages >25% are not shown). ^*^p < 0.05 and ^**^p < 0.01, respectively, as determined by Fisher's test with false discovery rate adjustments. COG functional annotations and specific values are summarized in Table S1.

To analyze the functional content of the genome and the distribution of CDSs across the replicons, COG functional classification analysis was conducted for all proteins in the NH9 genome (Figure 2). In addition, the percentage of proteins assigned to COG categories in each replicon was compared between chromosomes and between plasmids (Table S1). A significant difference in functionality was observed between the replicons, with the main chromosome, Chr1, encoding proteins responsible for core cellular functions, including protein processing (class O), translational machinery (class J), DNA replication and repair (class L), amino acid metabolism (class E), and nucleotide metabolism (class F). In comparison, the smaller chromosome, Chr2, showed a functional bias toward cell motility (class N), transcription (class K), and energy metabolism (classes C, I, and Q), indicating that proteins encoded on Chr2 are mainly related to adaptation and survival. These biases are similar to those observed in other *Cupriavidus* genomes (Janssen et al., 2010; Wang X. et al., 2015). As expected, the two plasmids coded for a higher percentage of proteins involved in partitioning (class D) and plasmid replication (class L), as well as proteins of unknown function (class -), compared with the chromosomes (Figure 2 and Table S1), as has been reported previously (Leplae et al., 2006). The smaller plasmid, pENH91, uniquely coded for proteins involved in intracellular trafficking and secretion (class U), while the larger plasmid, pENH92, was not associated with any significantly different protein functions, but did show a functional bias toward energy metabolism, including amino acid, nucleotide, carbohydrate, coenzyme, and lipid metabolism (classes C, E, F, G, H, and I).

A *dnaA* homolog and three DnaA boxes [TT(A/T)TNCACA] (Schaper and Messer, 1995) with no or only one mismatch compared with the consensus were located around the putative replication origin (*oriV*) of Chr1 (Figure S1A). Chr2 contained both *repA* and *parAB* homologs and 20 iteron-like repeats that may be involved in RepA binding [CGC(A/T)GA(A/T)(A/T)(C/T)(A/C/G)GGT(A/T)CG(C/G) consensus sequences] (Figure S1B), indicating that the partitioning system of Chr2 may be plasmid-like. Chr2 also contained three DnaA boxes with only one mismatch compared with the consensus. These chromosomal replication initiation systems were identical to those of other *Cupriavidus* strains such as *C*. *necator* H16 (Pohlmann et al., 2006), *C*. *metallidurans* CH34 (Janssen et al., 2010), and *C*. *gilardii* CR3 (Wang X. et al., 2015).

Plasmid pENH91 harbored a *trfA* gene with 100% amino acid sequence identity to that of incompatibility (Inc) P-1β plasmid pA81 of *Achromobacter xylosoxidans* A8 (Jencova et al., 2008) (Figure S1C). Because Inc plasmid groups are classified based on the amino acid sequence of the replication initiation protein (Shintani et al., 2015), we propose that pENH91 is a member of the IncP-1β plasmid family. Using a BLASTN analysis of ArcWithColor (Ohtsubo et al., 2008) with the following parameters: wordsize, 21; *E*-value, < 1e^−5^; and filter query sequence, F, we determined that 74,985 bp (97.2%) of the 77,172-bp pENH91 nucleotide sequence showed 100% identity to the sequence of the corresponding part of pA81 (98,192 bp). In our previous study, we proposed that pENH91 is an IncP-1 group plasmid based on incompatibility test results (Ogawa and Miyashita, 1995). Therefore, the results of the current study support the previous classification of pENH91 as an IncP-1 plasmid. Five putative TrfA-binding sites (iterons) (Norberg et al., 2014) [(A/C/G)(A/C/T)GCCCC(C/T)CA(A/T)GTGTCA consensus sequences] were located between hypothetical protein-coding gene (BJN34_0385) and addiction module antitoxin-coding gene (BJN34_37215) (Figure S1C), suggesting that *oriV* was located in this region.

pENH92 contained both *repB* and *parAB* and at least three DnaA boxes with no, one, or two mismatches compared with the consensus (Figure S1D). Interestingly, the predicted RepB and ParAB protein sequences showed greater similarity to those of pOLGA1 from *Burkholderia* sp. OLGA172 (Ricker et al., 2016) and pBN1 from *Paraburkholderia aromaticivorans* BN5 (Lee and Jeon, 2018) (63.8–82.8% amino acid identity) than to those of pBB1 from *C*. *necator* N-1 (Poehlein et al., 2011) and pRALTA from *C*. *taiwanensis* LMG19424 (Amadou et al., 2008) (39.3–71.9% amino acid identity). As mobile genetic elements (integrase and transposase) were identified either side of *repB* and *parAB* in pENH92 (Figure S1D), this region may have been acquired via horizontal transfer.

### Capacity of NH9 to Catabolize Aromatic Compounds

**Figure 3:**
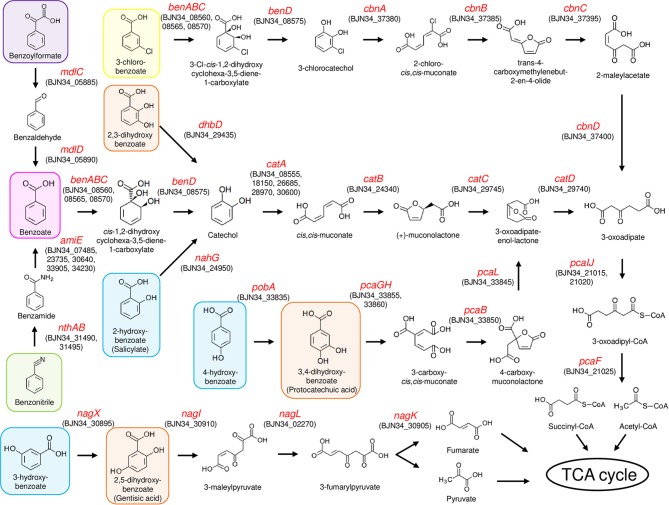
Aromatic compound degradation pathways in NH9. Enzyme-encoding genes are shown by red text, including locus tag(s). 3-chlorobenzoate, benzoate, benzonitrile, and benzoylformate are shaded in yellow, magenta, green, and purple, respectively. Hydroxybenzoate and dihydroxybenzoate are shaded in blue and orange, respectively.

To analyze the ability of NH9 to catabolize recalcitrant compounds other than 3-CB, the metabolic pathway for aromatic compound degradation was reconstructed using the KEGG database. Pathway analysis suggested that NH9 should be able to completely degrade benzoate, 2-, 3-, or 4-hydroxybenzoate, 2,3-, 2,5-, or 3,4-dihydroxybenzoate, benzoylformate, and benzonitrile (Table 2 and Figure 3). The catabolic capacity of NH9 was similar to that of other *Cupriavidus* strains; however, it lacked the complete set of phenol-degrading genes that are found in a number of other strains (Figure S2 and Table S2).

**Table 2:** Putative genes of *C*. *necator* NH9 involved in degradation of aromatic and relative compounds.

**Compound name**	**Gene designation^a^**	**K number**	**EC number**	**Definition^a^**	**Annotated genes^b^^,^^c^**
Benzonitrile	*nthA*	K01721	EC:4.2.1.84	Nitrile hydratase alpha subunit	BJN34_31490 (C2)
	*nthB*	K20807	EC:4.2.1.84	Nitrile hydratase beta subunit	BJN34_31495 (C2)
	*amiE*	K01426	EC:3.5.1.4	Amidase	BJN34_07485 (C1), 23735 (C2), 30640 (C2), 33905 (C2), 34230 (C2)
Benzoylformate	*mdlC*	K01576	EC:4.1.1.7	Benzoylformate decarboxylase	BJN34_05885 (C1)
	*mdlD*	K00141	EC:1.2.1.28	Benzaldehyde dehydrogenase	BJN34_05890 (C1)
Benzoate	*benA*	K05549	EC:1.14.12.10	Benzoate 1,2-dioxygenase alpha subunit	BJN34_08560 (C1)
	*benB*	K05550	EC:1.14.12.10	Benzoate 1,2-dioxygenase beta subunit	BJN34_08565 (C1)
	*benC*	K05784	EC:1.14.12.10	Benzoate 1,2-dioxygenase reductase component	BJN34_08570 (C1)
	*benD*	K05783	EC:1.3.1.25	1,6-dihydroxycyclohexa-2,4-diene−1-carboxylate dehydrogenase	BJN34_08575 (C1)
2-hydroxybenzoate(Salicylate)	*nahG*	K00480	EC:1.14.13.1	Salicylate hydroxylase	BJN34_24950 (C2)
3-hydroxybenzoate	*nagX*	K22270	EC:1.14.13.24	3-hydroxybenzoate 6-monooxygenase	BJN34_30895 (C2)
	*nagI*	K00450	EC:1.13.11.4	Gentisate 1,2-dioxygenase	BJN34_30910 (C2)
	*nagL*	K01801	EC:5.2.1.4	Maleylpyruvate isomerase	BJN34_02270 (C1)^d^
	*nagK*	K16165	EC:3.7.1.20	Fumarylpyruvate hydrolase	BJN34_30905 (C2)
4-hydroxybenzoate	*pobA*	K00481	EC:1.14.13.2	4-hydroxybenzoate 3-monooxygenase	BJN34_33835 (C2)
	*pcaG*	K00448	EC:1.13.11.3	Protocatechuate 3,4-dioxygenase, alpha subunit	BJN34_33855 (C2)
	*pcaH*	K00449	EC:1.13.11.3	Protocatechuate 3,4-dioxygenase, beta subunit	BJN34_33860 (C2)
	*pcaB*	K01857	EC:5.5.1.2	3-carboxy-*cis*,*cis*-muconate cycloisomerase	BJN34_33850 (C2)
	*pcaL*	K14727	EC:4.1.1.44	4-carboxymuconolactone decarboxylase	BJN34_33845 (C2)
2,3-dihydroxybenzoate	*dhbD*	K14333	EC:4.1.1.46	2,3-dihydroxybenzoate decarboxylase	BJN34_29435 (C2)
3-chlorocatechol	*cbnA*	K15253	EC:1.13.11.-	Chlorocatechol 1,2-dioxygenase	BJN34_37380 (p1)
	*cbnB*	K01860	EC:5.5.1.7.	Chloromuconate cycloisomerase	BJN34_37385 (p1)^d^
	*cbnC*	K01061	EC:3.1.1.45	Dienelactone hydrolase	BJN34_37395 (p1)
	*cbnD*	K00217	EC:1.3.1.32	Maleylacetate reductase	BJN34_37400 (p1)^d^
Catechol	*catA*	K03381	EC:1.13.11.1	Catechol 1,2-dioxygenase	BJN34_08555 (C1), 18150 (C1), 26685 (C2), 28970 (C2), 30600 (C2)
	*catB*	K01856	EC:5.5.1.1	Muconate cycloisomerase	BJN34_24340 (C2)
	*catC*	K03464	EC:5.3.3.4	Muconolactone isomerase	BJN34_29745 (C2)
	*catD*	K01055	EC:3.1.1.24	3-oxoadipate enol-lactonase	BJN34_29740 (C2)
3-oxoadipate	*pcaI*	K01031	EC:2.8.3.6	3-oxoadipate CoA-transferase, alpha subunit	BJN34_21015 (C2)
	*pcaJ*	K01032	EC:2.8.3.6	3-oxoadipate CoA-transferase, beta subunit	BJN34_21020 (C2)
	*pcaF*	K00632	EC:2.3.1.16	3-oxoadipyl-CoA thiolase	BJN34_21025 (C2)^d^

a *Gene designation and definition from KEGG annotation were manually modified*.

b *Genes listed in this table were manually selected*.

c *Gene locations were shown as below; C1, Chromosome1; C2, Chromosome 2; p1, pENH91*.

d *2nd best hit*.

Genes of NH9 involved in the decomposition of the compounds described above were mostly located on chromosome, especially Chr2, although the *ben* genes, coding for the key elements of benzoate and 3-CB degradation, were located on Chr1 (Table 2). Almost all of the genes involved in the dissimilation of aromatic compounds were found within clusters, although genes required for the degradation of 3-hydroxybenzoate and catechol were dispersed between Chr1 (*nagL* and *catA*) and Chr2 (*catAB*) (Table 2). BLASTN analysis showed that *nagL* homolog (BJN34_30900) was located between *nagX* (BJN34_30895) and *nagK* (BJN34_30905) on Chr2, indicative of an operon structure.

A *cat* gene cluster containing all the three genes, *catA, catB*, and *catC*, for catechol degradation was not observed in the NH9 genome, suggesting that the *cat* genes were not acquired at the same time. In various Gram-negative bacteria (e.g., *P. putida* and *Pseudomonas resinovorans*), *cat* genes form an operon including *catR*, a LysR-type transcriptional regulator. Two *catR* homologs were identified upstream of *catA* (BJN34_08555) on Chr1 and *catB* (BJN34_24340) on Chr2, respectively, in the NH9 genome. Because catechol degradation is one of the central pathways in the metabolism of a variety of aromatic compounds (Broderick, 1999), the acquisition of the various *cat* genes is intriguing from an evolutionary standpoint in terms of the ability of NH9 to degrade aromatic compounds. Interestingly, several putative CatA-coding genes were present on chromosome 1 and 2 (Table 2). Catechol 1,2-dioxygenase, the product of *catA*, cleaves the aromatic ring of catechol between two hydroxyl groups (intradiol cleavage) (Figure 3). Other homologous ring-cleaving dioxygenase enzymes such as chlorocatechol dioxygenase and protocatechuate 3,4-dioxygenase catalyze similar reactions (Neidle et al., 1988), with this aromatic ring cleavage recognized as a critical step in the complete degradation of chlorinated or non-chlorinated aromatic compounds by soil bacteria (Harwood and Parales, 1996; Reineke, 1998; Broderick, 1999).

In summary, strain NH9 shared several putative pathways for aerobic degradation of aromatic compounds with other *Cupriavidus* strains (Table 2, Figure S2, and Table S2). The characteristic feature of strain NH9 is its ability to degrade 3-CB which is an intermediate metabolite of the degradation of PCBs (Reineke and Knackmuss, 1988). Benzoate, hydroxybenzoates and dihydroxybenzoates are intermediate metabolites of the degradation of plant-derived compounds and polycyclic aromatic hydrocarbons (PAHs) (Seo et al., 2009; Wang J. Y. et al., 2015), suggesting that NH9 may be adapted for catabolism of those simple aromatic compounds in the soil environment.

### Phylogenetic Analyses

In order to understand the genetic characteristics of the predicted degradation ability of the strain NH9 among related bacteria, precise taxonomic identification of NH9 was required. The necessity also arose from an intertwined history of the genera *Cupriavidus* with several other β-proteobacteria. In particular, there have been several taxonomic problems within the genera *Cupriavidus* and *Ralstonia* because of their genomic diversity and similarities (Vandamme and Coenye, 2004). For instance, *R*. *pickettii* DTP0602 and *Ralstonia* sp. PBA were proposed to belong to the genus *Cupriavidus* (Zhang and Qiu, 2016; Kim and Gan, 2017). It is possible that there are other bacteria currently belonging to these two genera that should be reclassified.

Previous studies of related bacterial genera suggested relationship between the degradation ability of aromatic compounds and the phylogenetic location (Harwood and Parales, 1996; Perez-Pantoja et al., 2012). Thus, to understand first the distribution of aromatic degradation capabilities in the genus *Cupriavidus* and *Ralstonia*, orthologous genes and potential abilities to degrade aromatic compounds in selected *Cupriavidus* and *Ralstonia* strains were surveyed (Figure S2 and Table S2). These results indicated that the putative phenotype of degradation ability and taxonomic classification of those strains were not consistent absolutely, although there is a tendency that *Ralstonia* strains lack genes for degradation of several aromatic compounds which are present in most *Cupriavidus* strains. The above results led us to examining the genomes of all strains of the genera *Cupriavidus* and *Ralstonia* whose complete or draft genome sequences are available from the NCBI database in order to perform genome-based phylogenetic comparison. As a result, we propose reclassification of several strains belonging to these two genera. All *Cupriavidus* and *Ralstonia* strains examined in this study are shown in Table 3 and Table S3.

**Table 3:** *Cupriavidus* and *Ralstonia* strains used in this study.

**Genus**	**Species**	**Strain**	**Phylotype sequevar**	**# assembly accession**	**ANI cluster**	**TNA cluster**
*Cupriavidus*	*alkaliphilus*	ASC-732^a^	–	GCF_900094595.1	3	B
	*basilensis*	4G11	–	GCF_000832305.1	7	E
		KF708	–	GCF_000876015.1	9	C
		OR16	–	GCF_000243095.1	7	E
	*gilardii*	CR3	–	GCF_001281465.1	10	I
		JZ4	–	GCF_001658125.1	10	I
	*metallidurans*	CH34^a^	–	GCF_000196015.1	16	L
		Ni-2	–	GCF_002944765.1	16	L
		H1130	–	GCF_000496715.1	16	L
		NA1	–	GCF_000709025.1	16	L
		NA4	–	GCF_000709045.1	16	L
		NBRC 101272	–	GCF_001598775.1	16	L
		NDB3NO24	–	GCF_001543455.1	16	L
		NE12	–	GCF_000709065.1	16	L
	*nantongensis*	X1^a^	–	GCF_001598055.1	3	B
	*necator*	H16	–	GCF_000009285.1	1	D
		N-1^a^	–	GCF_000219215.1	1	D
		NH9	–	GCF_002011925.2	1	D
		A5-1	–	GCF_000744095.1	2	G
		NBRC 102504	–	GCF_001598755.1	1	D
		PHE3-6	–	GCF_001853325.1	1	D
	*oxalaticus*	NBRC 13593	–	GCF_001592245.1	4^c^	B^c^
	*pauculus*	KF709	–	GCF_000974605.1	15^c^	K^c^
		UM1	–	GCF_002858765.1	14	J
	*pinatubonensis*	JMP134	–	GCF_000203875.1	6^c^	K^c^
	*taiwanensis*	LMG19424^a^	–	GCF_000069785.1	3	B
		STM 6018	–	GCF_000472465.1	3	B
		STM6070	–	GCF_000372525.1	3	B
	sp.	USMAA1020	–	GCF_001854325.1	11	A
		USMAA2-4	–	GCF_001854305.1	11	A
		USMAHM13	–	GCF_001854285.1	11	A
		amp6	–	GCF_000426345.1	4	G
		BIS7	–	GCF_000292345.1	17^c^	K^c^
		D384	–	GCF_001652915.1	14	J
		GA3-3	–	GCF_000389805.1	1	D
		HMR-1	–	GCF_000319775.1	16	L
		HPC(L)	–	GCF_000307735.3	10	I
		IDO	–	GCF_000812465.1	5	G
		OV038	–	GCF_900112215.1	12	H
		OV096	–	GCF_900115455.1	12	H
		SHE	–	GCF_000812445.1	16	L
		SK-3	–	GCF_000611145.1	8	F
		SK-4	–	GCF_000611125.1	1	D
		UYPR2.512	–	GCF_000379565.1	1	D
		WS	–	GCF_000395345.1	9	C
		YR651	–	GCF_900101625.1	13^c^	K^c^
*Ralstonia*	*insidiosa*	ATCC 49129	–	GCF_001663855.1	20	N
		FC1138	–	GCF_001653935.1	20	N
		WCHRI065162	–	GCF_002939165.1	20	N
		WCHRI065437	–	GCF_002939035.1	20	N
	*mannitolilytica*	SN82F48	–	GCF_000954135.1	19	O
		SN83A39	–	GCF_001628775.1	19	O
		GML-Rals1-TR	–	GCF_002863525.1	19	O
		MRY14-0246	–	GCF_000953875.1	19	O
		WCHRM065694	–	GCF_002939115.1	19	O
		WCHRM065837	–	GCF_002939145.1	19	O
	*pickettii*	12D	–	GCF_000023425.1	18	M
		12J	–	GCF_000020205.1	18	M
		DTP0602	–	GCF_000471925.1	2	G
		FDAARGOS_410	–	GCF_002393485.1	19	O
		52	–	GCF_002849525.1	18	M
		5_7_47FAA	–	GCF_000165085.1	18	M
		ATCC 27511^a^	–	GCF_000743455.1	18	M
		CW2	–	GCF_000607185.1	18	M
		H2Cu2	–	GCF_001699795.1	18	M
		H2Cu5	–	GCF_001699815.1	18	M
		ICMP-8657	–	GCF_002516395.1	18	M
		NBRC 102503	–	GCF_001544155.1	18	M
		OR214	–	GCF_000372665.1	18	M
		SSH4	–	GCF_000607165.1	18	M
	*solanacearum*	GMI1000	I-18	GCF_000009125.1	23	P
		CQPS-1	I	GCF_002220465.1	23	P
		EP1	I	GCF_001891105.1	23	P
		FJAT-91	I	GCF_002155245.1	23	P
		FJAT-1458	I	GCF_001887535.1	23	P
		FQY_4	I	GCF_000348545.1	23	P
		Rs-09-161	I	GCF_000671335.1	23	P
		Rs-10-244	I	GCF_000671315.1	23	P
		P781	I-14	GCF_001644865.1	23	P
		UW757	I-14	GCF_001645725.1	23	P
		CaRs-Mep	I	GCF_001855495.1	23	P
		SD54	I	GCF_000430925.2	23	P
		Y45	I	GCF_000223115.1	23	P
		UW25^a^	IIA-7	GCF_002251695.1	25	P
		Grenada 9-1	IIA-6	GCF_000825845.1	25	P
		B50	IIA-24	GCF_000825785.1	25	P
		IBSBF1900	IIA-24	GCF_001373275.1	25	P
		CIP120	IIA-38	GCF_001644795.1	25	P
		P597	IIA-38	GCF_001644805.1	25	P
		UW551	IIB-1	GCF_002251655.1	25	P
		UY031	IIB-1	GCF_001299555.1	25	P
		IBSBF1503	IIB-4	GCF_001587155.1	25	P
		Po82	IIB-4	GCF_000215325.1	25	P
		UW163	IIB-4	GCF_001587135.1	25	P
		CFBP3858	IIB-1	GCF_001373335.1	25	P
		IPO1609	IIB-1	GCF_001050995.1	25	P
		NCPPB 909	IIB-1	GCF_000710695.1	25	P
		POPS2	IIB-1	GCF_000750585.1	25	P
		RS2	IIB-1	GCF_001373295.1	25	P
		UW365	IIB-1	GCF_001696865.1	25	P
		UW491	IIB-1	GCF_001696845.1	25	P
		NCPPB 282	IIB-2	GCF_000750575.1	25	P
		CFBP1416	IIB-3	GCF_000825925.1	25	P
		CIP417	IIB-3	GCF_000825825.1	25	P
		MolK2	IIB-3	GCF_000212635.3	25	P
		CFBP6783	IIB-4	GCF_001644815.1	25	P
		P673	IIB-4	GCF_000525615.1	25	P
		UW179	IIB-4	GCF_000825805.1	25	P
		23-10BR	IIB-27	GCF_000749995.1	25	P
		CFBP7014	IIB-59	GCF_001373255.1	25	P
		CFIA906	IIB	GCF_000710135.2	25	P
		CMR15	III	GCF_000427195.1	23	P
		CFBP3059	III-48	GCF_001644855.1	23	P
		PSI07^a^	IV	GCF_000283475.1	24	P
		KACC10709	NA^b^	GCF_001708525.1	23	P
		KACC 10722	NA^b^	GCF_001586135.1	24	P
		OE1-1	NA^b^	GCF_001879565.1	23	P
		RS 488	NA^b^	GCF_002501565.1	25	P
		RS 489	NA^b^	GCF_002549815.1	25	P
		RSCM	NA^b^	GCF_002894285.1	23	P
		SEPPX05	NA^b^	GCF_002162015.1	23	P
		YC40-M	NA^b^	GCF_001663415.1	23	P
		58_RSOL	NA^b^	GCF_001065525.1	20	N
		GEO_6	NA^b^	GCF_002894765.1	25	P
		GEO_55	NA^b^	GCF_002894845.1	25	P
		GEO_57	NA^b^	GCF_002029885.1	25	P
		GEO_81	NA^b^	GCF_002894785.1	25	P
		GEO_96	NA^b^	GCF_002029895.1	25	P
		GEO_99	NA^b^	GCF_002029865.1	25	P
		GEO_230	NA^b^	GCF_002894795.1	25	P
		GEO_304	NA^b^	GCF_002894775.1	25	P
		PSS4	NA^b^	GCF_001876985.1	23	P
		PSS190	NA^b^	GCF_001870825.1	23	P
		PSS216	NA^b^	GCF_001876975.1	23	P
		PSS1308	NA^b^	GCF_001870805.1	23	P
		RD15	NA^b^	GCF_001854265.1	23	P
		Rs-T02	NA^b^	GCF_001484095.1	23	P
		UTT-25	NA^b^	GCF_002930085.1	23	P
		UW24	NA^b^	GCF_001696855.1	25	P
		UW181	NA^b^	GCF_001373315.1	25	P
	sp.	25mfcol4.1	–	GCF_900104095.1	14	J
		5_2_56FAA	–	GCF_000227255.2	18	M
		A12	–	GCF_000801955.1	22	N
		AU12-08	–	GCF_000442475.1	20	N
		MD27	–	GCF_001078575.1	21	N
		NFACC01	–	GCF_900115545.1	18	M
		NT80	–	GCF_001485395.1	20	N
		PBA	–	GCF_000272025.1	NA^b, c^	K^c^
		UNC404CL21Col	–	GCF_000620465.1	18	M
		UNCCL144	–	GCF_900099845.1	18	M

a *Type strains that are proposed in List of prokaryotic names with standing in nomenclature (http://www.bacterio.net/index.html)*.

b *Not assigned*.

c *Contradictory clusterings between ANI analysis and TNA*.

**Figure 4:**
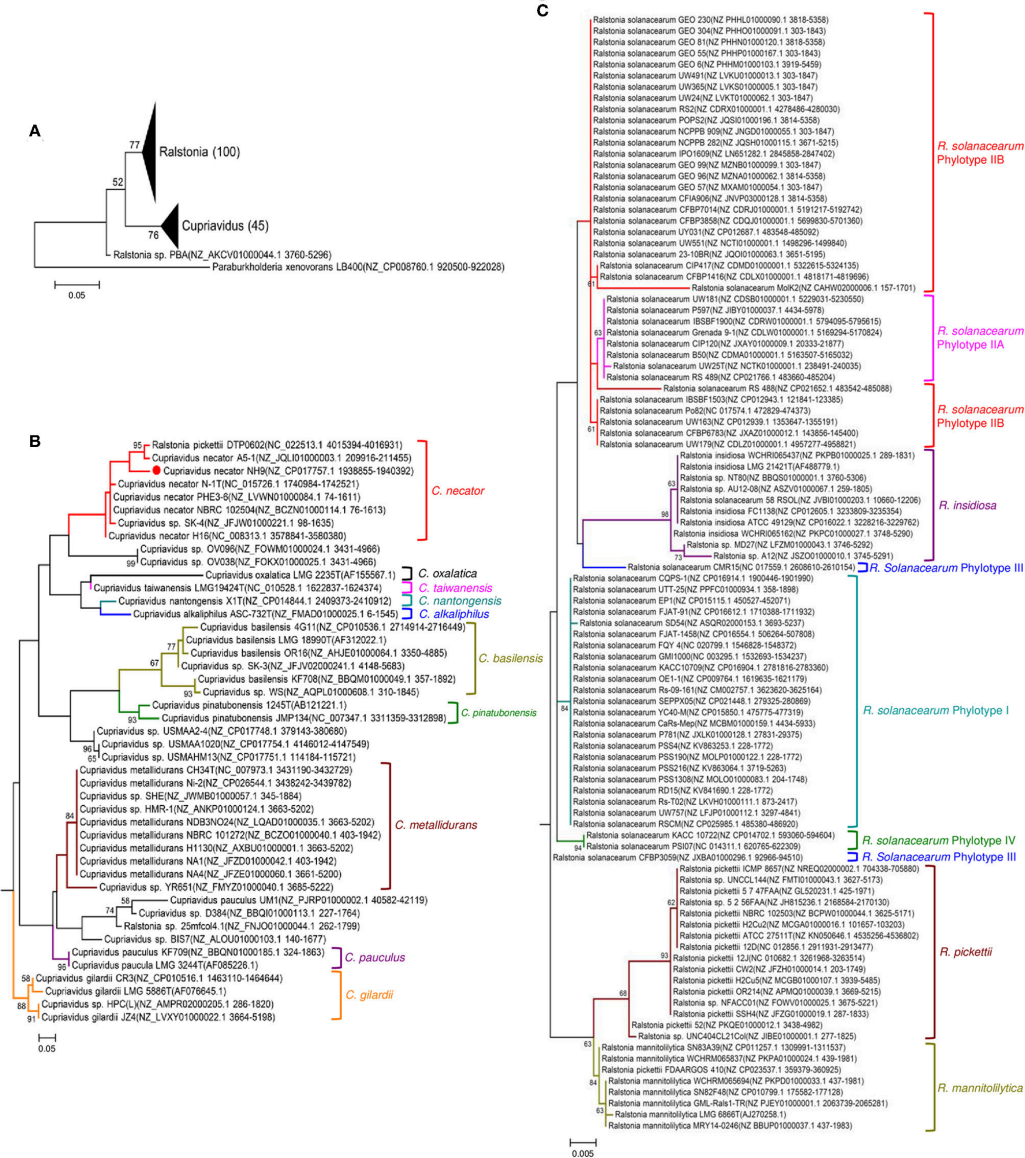
Maximum likelihood trees showing the phylogeny of the genera *Cupriavidus* and *Ralstonia* based on 16S rRNA gene sequences. Phylogenetic trees generated for both genera **(A)**, the genus *Cupriavidus* **(B)**, and the genus *Ralstonia* **(C)**. Full-length 16S rRNA gene sequences were aligned and used in the phylogenetic analysis (see section Materials and Methods for detail). Proposed type-strains are designated with a “T.” Strain NH9 is marked with a red circle. Bootstrap values are represented at the branching points (only values >50% are shown).

Phylogenetic relationships between the genera *Cupriavidus* and *Ralstonia*, including strain NH9 and several type strains, were inferred based on 16S rRNA gene sequences (Figure 4). A clear separation of the genus *Cupriavidus* from the genus *Ralstonia* could be seen in the phylogenetic tree (Figure 4A). Overall, only *Ralstonia* sp. PBA was not grouped into either of the clades. The tree generated for the genus *Cupriavidus* showed a number of clades and included two strains currently classified as *Ralstonia* (Figure 4B). *R*. *pickettii* DTP0602 clustered into the *C*. *necator* clade (type strain *C*. *necator* N-1) and *Ralstonia* sp. 25mfcol4.1 was also included in a clade consisting only of *Cupriavidus* sp. strains. *C. pinatubonensis* JMP134 and *C*. *pinatubonensis* 1245^T^ formed a homogeneous group with very high similarity (99.6%), as previously reported (Sato et al., 2006). Four different species, *C*. *oxalaticus, C*. *taiwanensis, C*. *nantongensis*, and *C*. *alkaliphilus*, were contained in one clade, reflecting the high level of similarity among the 16S rRNA gene sequences of these species (>97.2%). In particular, *C*. *taiwanensis* LMG19424^T^, *C*. *nantongensis* X1^T^, and *C*. *alkaliphilus* ASC-732^T^ showed very high sequence similarities (>99.1%), as previously reported (Sun et al., 2016). Strain NH9 shared 99.2% 16S rRNA nucleotide sequence similarity with *C*. *necator* N-1^T^ and, as expected, was categorized into the *C*. *necator* clade. The genus *Ralstonia* formed two large clades, one consisting of *R*. *pickettii* (type strain *R*. *pickettii* ATCC 27511) and *R*. *mannitolilytica* and the other comprising *R*. *insidiosa* and *R*. *solanacearum* (type strain *R*. *solanacearum* UW25) (Figure 4C). However, the *R*. *solanacearum* clade exhibited aberrant branching; for example, strains CMR15 and CFBP3059, which belong to phylotype III, did not form a clade. Additionally, the *R*. *insidiosa* clade was included within the *R*. *solanacearum* clade.

**Figure 5:**
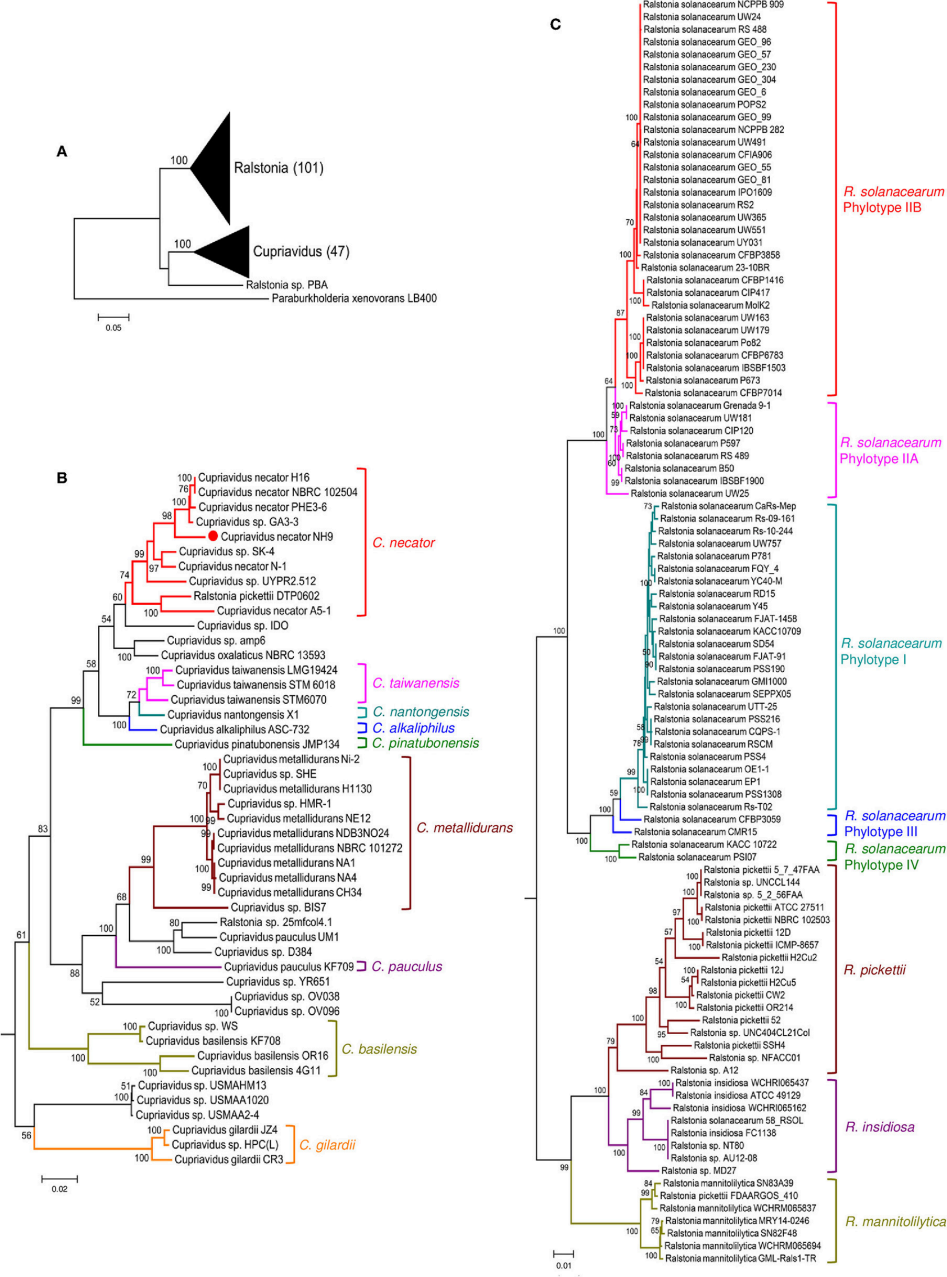
Maximum likelihood phylogenetic trees constructed using concatenated *atpD, leuS, rplB*, and *gyrB* nucleotide sequences from strains belonging to the genera *Cupriavidus* and *Ralstonia*. Phylogenetic trees generated for both genera **(A)**, the genus *Cupriavidus* **(B)**, and the genus *Ralstonia* **(C)**. The final dataset contained 6,333 positions (see section Materials and Methods for details). Strain NH9 is marked with a red circle. Bootstrap values are represented at the branching points (only values >50% are shown).

16S rRNA gene sequence-based phylogenetic analysis is a basic approach used in prokaryotic taxonomy; however, it cannot provide sufficient resolution to discriminate sequences to the species level. Because MLSA can help to resolve phylogenetic relationships at the genus and species levels, we also constructed a MLSA-based phylogenetic tree using four single-copy housekeeping genes, *atpD, leuS, rplB*, and *gyrB*, obtained from 45 *Cupriavidus* and 104 *Ralstonia* strains (Figure 5). *leuS, rplB*, and *gyrB* were also used in a recent MLSA study of *R. solanacearum* (Zhang and Qiu, 2016). The concatenated gene sequence-based phylogenetic tree showed broadly similar patterns to the 16S rRNA-based phylogeny and all clades were clearly resolved, although *Ralstonia* sp. PBA was grouped within the same clade as the genus *Cupriavidus* (Figure 5A). This branching of strain PBA was consistent with a previously reported maximum likelihood tree (Kim and Gan, 2017). Additionally, the phylogenetic positions of *Cupriavidus* sp. BIS7, *Cupriavidus* sp. YR651, and *Ralstonia* sp. A12 differed compared with the 16S rRNA-based tree (Figures 5B,C). As has been reported previously, a clade consisting of the *R*. *solanacearum* strains showed rational branching (Zhang and Qiu, 2016). Furthermore, the *R*. *insidiosa* clade was separated from the *R*. *solanacearum* clade (Figure 5C). Bootstrap values in the concatenated gene tree were significantly higher than those in the 16S rRNA gene-based tree, indicating that the observed branching patterns were reliable.

### Average Nucleotide Identity Analysis

**Figure 6:**
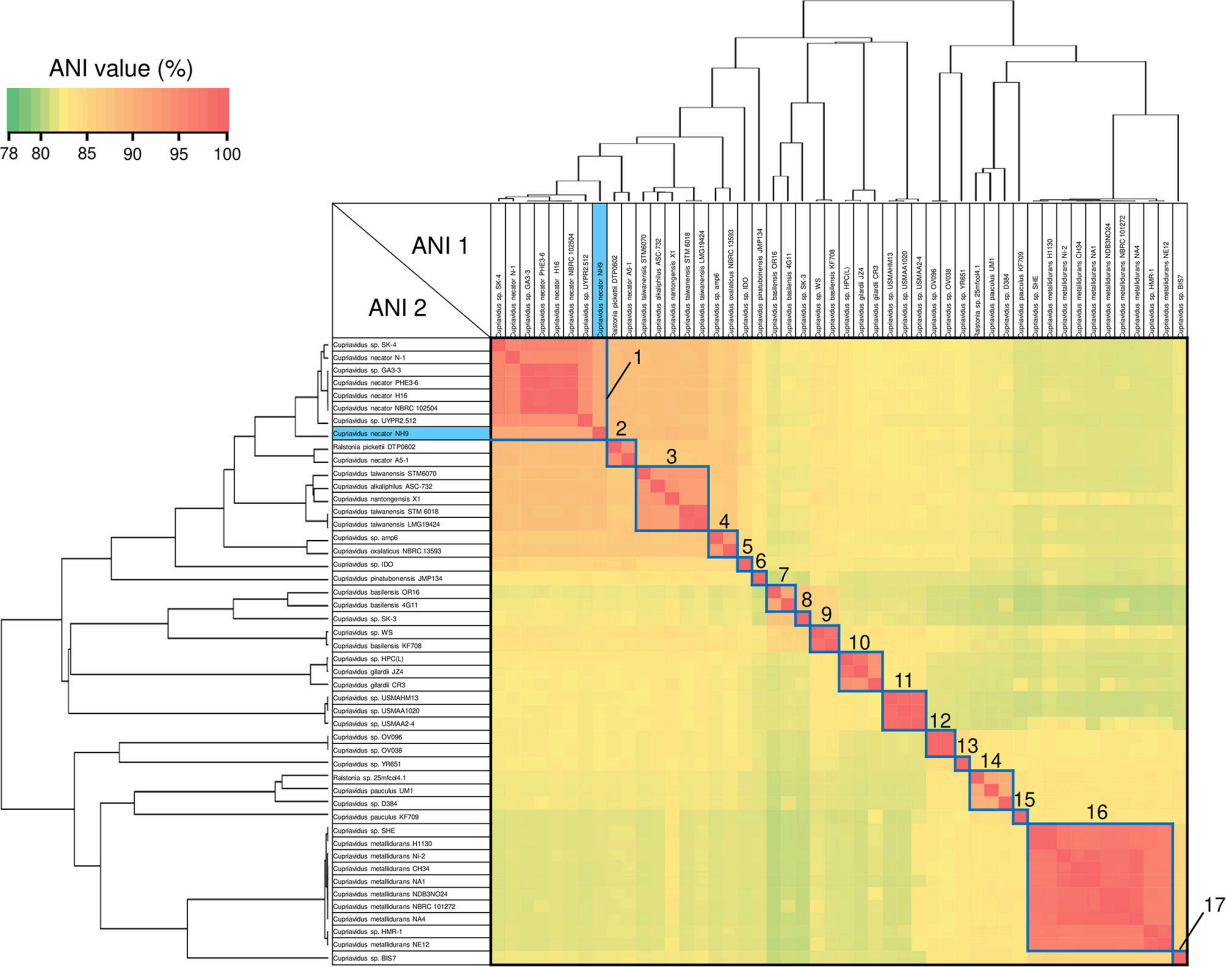
Heatmap and dendrogram of average nucleotide identity (ANI) values of the 48 putative *Cupriavidus* genomes. Strains with ANI values >90% are shown within blue squares (see Table 3 and Table S4 for a description of cluster designations). All squares were assigned numbers from 1 to 17.

**Figure 7:**
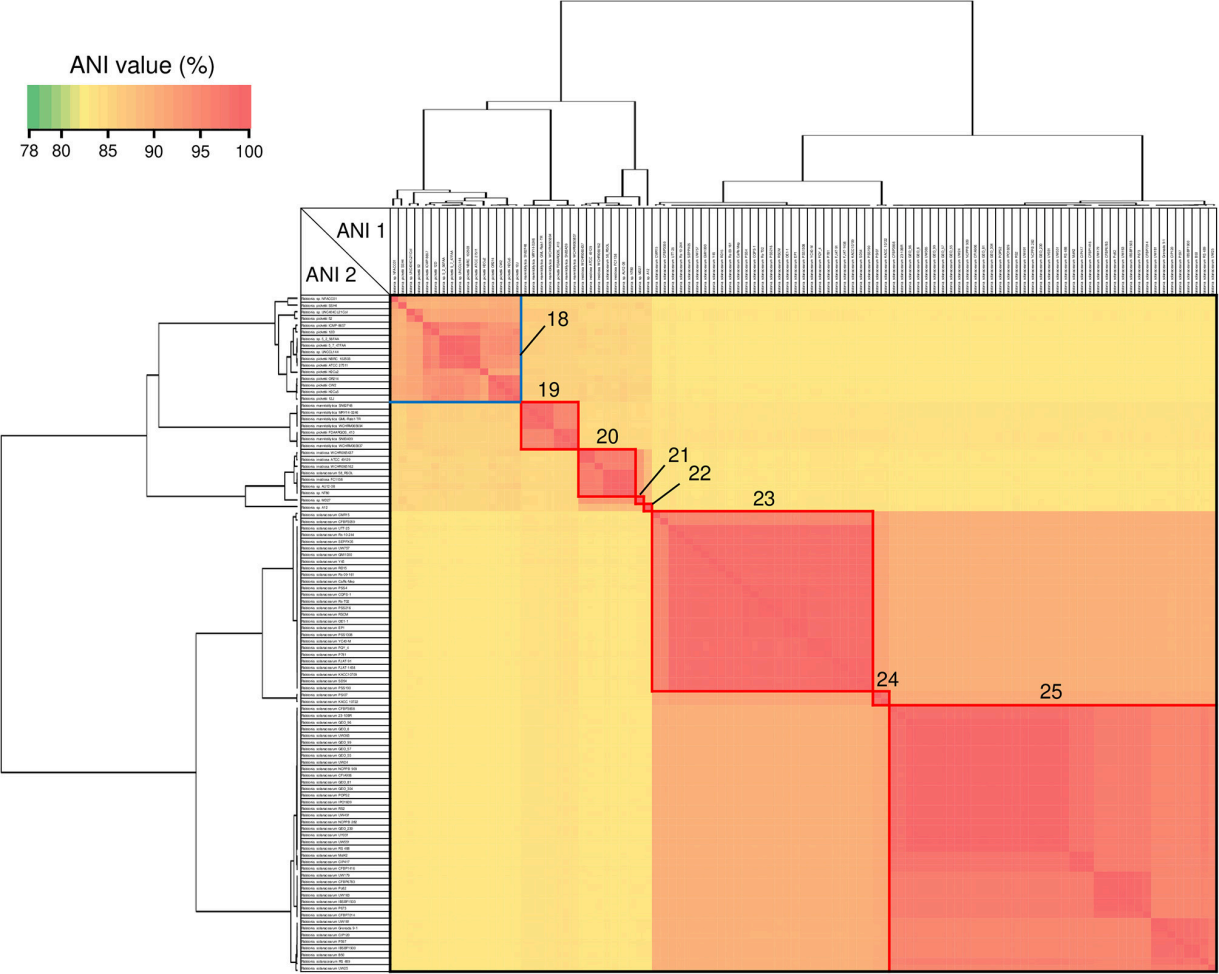
Heatmap and dendrogram of average nucleotide identity (ANI) values of the 101 putative *Ralstonia* genomes. Strains with ANI values >90 and >95% are shown within blue and red squares, respectively (see Table 3 and Table S4 for a description of cluster designations). All squares were assigned a number from 18 to 25.

ANI analysis is usually used for bacterial species delineation along with MLSA (Goris et al., 2007). To assess genome similarities, ANI analysis was performed using all downloaded *Cupriavidus* and *Ralstonia* genome sequences (Figures 6, 7, and Table S4). Clusters determined by ANI analysis are described in Table 3. The ANI cut-off value for species distinction is generally 95–96% (Richter and Rossello-Mora, 2009; Kim et al., 2014; Ciufo et al., 2018). While this threshold value was applicable to many *Ralstonia* strains examined in this study (Table S4), we propose that in the case of the genus *Cupriavidus* and the species *R*. *pickettii*, 90% is a more reasonable threshold considering the results of ANI, phylogenetic analyses, and TNA. For instance, the ANI score of strain NH9 was 91.16% (ANI1 and 2) when compared with *C*. *necator* N-1^T^ (Table S4), which would suggest that NH9 does not belong to the species *C*. *necator* when using a standard ANI threshold value. Likewise, some of the *R*. *pickettii* strains were not correctly grouped at a 95–96% cut-off value. Richter and Rossello-Mora reported that at ANI values below 90%, taxonomic differences became more evident, suggesting that ANI values above 90% produce more robust results (Richter and Rossello-Mora, 2009). Furthermore, it has been discovered that several genera contain species with non-standard ANI cut-off points, which is thought to reflect species diversity (Kim et al., 2014; Ciufo et al., 2018). As noted above, the genus *Cupriavidus* and the species *R*. *pickettii* are very diverse. The relationships between strains categorized at a 90% ANI cut-off were almost identical to the groupings identified by phylogenetic analysis and TNA. Therefore, we propose relaxation of the ANI cut-off value from 95–96 to 90% for the genus *Cupriavidus* and the species *R*. *pickettii*.

We also performed additional DNA comparisons whereby the two main replicons from each of the complete genome-sequenced *Cupriavidus* and *Ralstonia* strains were, respectively, compared with those from all of the other strains. It was expected that the ANI cut-off values produced from the comparisons between main replicons and between second replicons would be dramatically improved relative to the original results; however, this was not the case (data not shown). This result thus seems to endorse the validity of ANI analysis using the whole-genome sequence of each strain.

Species grouped with ANI values >90 and 95% are shown in blue and red boxes, respectively, in Figures 6, 7. *Cupriavidus* and *Ralstonia* strains formed 17 and 8 groups, respectively, indicating greater diversity within the genus *Cupriavidus* compared with *Ralstonia*. Only *Ralstonia* sp. PBA did not share similarity with either genus, as was also observed in the 16S rRNA gene-based phylogeny (Table S4). Interestingly, group 3 included three different species (ANI values >92%): *C*. *alkaliphilus, C*. *nantongensis*, and *C*. *taiwanensis*. This is not surprising given the abovementioned high degree of 16S rRNA nucleotide sequence similarity (>99.1%) between the three species. While ANI-based groupings were consistent with the MLSA-based phylogenetic relationships, some variations in the grouping patterns were observed. *C*. *necator* A5-1 and *R*. *pickettii* DTP0602 (group 2) were separated from the species *C*. *necator* (group 1), while the members of group 9, *C*. *basilensis* KF708 and *Cupriavidus* sp. WS, were distinguished from the species *C*. *basilensis* (group 7). *Cupriavidus* sp. BIS7 (group 17), *Ralstonia* sp. MD27 (group 21), and *Ralstonia* sp. A12 (group 22), which were categorized into the *C*. *metallidurans, R*. *insidiosa*, and *R*. *pickettii* clades, respectively, in the MLSA-based phylogeny, were separated from their respective groups in this analysis. Further, *Ralstonia* sp. A12 showed higher similarity to *R*. *insidiosa* (group 20) than to *R*. *pickettii* (group 18). The species *R*. *solanacearum* formed three subgroups, consisting of phylotype I and III (group 23), phylotype IV (group 24), and phylotype II (group 25) strains, with an ANI value of 95%. It has previously been proposed that *R*. *solanacearum* should be classified into three species, *R*. *pseudosolanacearum* (phylotype I and III), *R*. *solanacearum* (phylotype II), and *R*. *syzygii* (phylotype IV) (Safni et al., 2014), which was validated by genomic, phylogenetic, and proteomic approaches (Prior et al., 2016; Zhang and Qiu, 2016). The result of ANI analysis supported this classification.

### Percentage of Conserved Proteins Analysis

**Figure 8:**
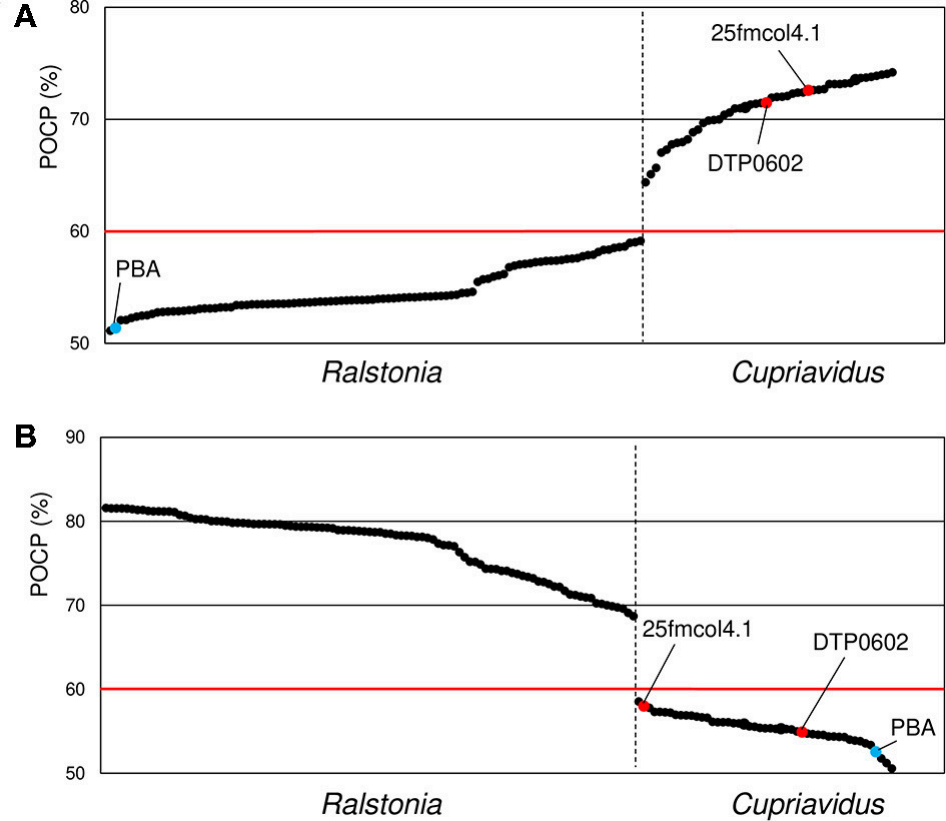
Comparison of average percentage of conserved proteins (POCP) values between *Cupriavidus* **(A)** and *Ralstonia* **(B)** named strains. *Ralstonia* group and *Cupriavidus* group strains are shown to the left and right of the vertical dotted bar, respectively. The two red dots indicate strains that are currently classified as *Ralstonia* but grouped into the genus *Cupriavidus* in this analysis. Blue dots indicate identical average POCP values when compared with *Cupriavidus* and *Ralstonia* strains. The red horizontal line indicates the genus boundary threshold (a POCP value of 60% was used in this study). Organism names are abbreviated as follows: 25fmcol4.1, *Ralstonia* sp. 25fmcol4.1; DTP0602, *R*. *pickettii* DTP0602; PBA, *Ralstonia* sp. PBA.

Phylogenetic analyses and ANI matrixes showed that two strains currently categorized as *Ralstonia, R*. *pickettii* DTP0602 and *Ralstonia* sp. 25fmcol4.1, belong to the genus *Cupriavidus*, and suggested that *Ralstonia* sp. PBA does not belong to either *Cupriavidus* or *Ralstonia*. However, while these analyses are useful for species delineation, they are not suitable for determining genera (Qin et al., 2014). Because the POCP method can provide comprehensive information for prokaryotic genus definition and delimitation, we performed POCP analysis for all *Cupriavidus* and *Ralstonia* strains (Figure 8). The POCP value threshold for a genus boundary is generally 50%; however, in the present case, we propose that a 60% POCP value is more rational. While most of the *Cupriavidus* and *Ralstonia* strains were correctly categorized into the appropriate genus, the two *Ralstonia* strains mentioned above showed same behavior as the *Cupriavidus* genus strains. This result confirmed that *R*. *pickettii* DTP0602 and *Ralstonia* sp. 25fmcol4.1 should be reclassified into the genus *Cupriavidus*. *Ralstonia* sp. PBA had POCP values of 51.4 and 52.6% when compared with *Cupriavidus* and *Ralstonia* strains, respectively, suggesting that it likely does not belong to either genus (Figure 8).

### Tetra-Nucleotide Analysis

**Figure 9:**
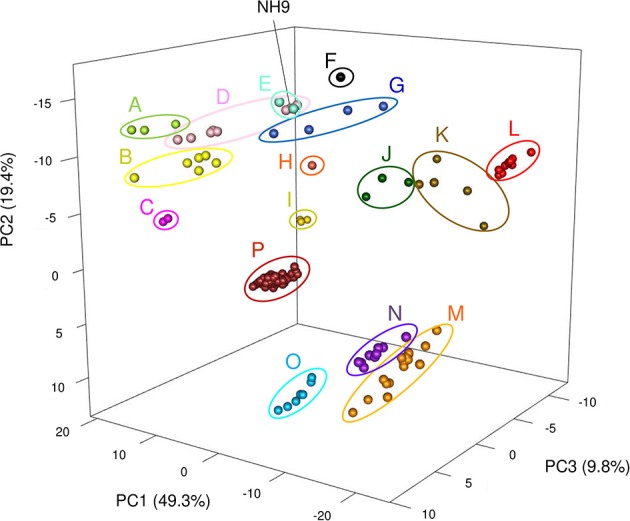
Three-dimensional plot of principal components analysis results for all 150 *Cupriavidus* and *Ralstonia* genomes. Differences in color between clusters indicates divergence using the first three principal components. Sixteen clusters (A to P) are shown (see Table 3 for a description of cluster assignments).

TNA and a PCA were also performed because tetra-nucleotide usage could be an alternative marker for clustering bacteria based on similarities in their genome sequence features (Richter and Rossello-Mora, 2009). These analyses showed that *Cupriavidus* strains formed 12 scattered clusters (clusters A–L) while *Ralstonia* strains formed only four clear clusters (clusters M–P), indicating the greater diversity of the *Cupriavidus* genome structure (Figure 9). Cluster assignments based on PCA are described in Table 3. Strain NH9 was categorized into Cluster D, consisting mainly of *C*. *necator* strains. PCA grouped *C*. *necator* A5-1 and *R*. *pickettii* DTP0602 into the same cluster (cluster G) as shown in the ANI matrix, with the addition of *Cupriavidus* sp. amp6 and *Cupriavidus* sp. IDO (Figure 9 and Table 3). Because *Cupriavidus* sp. amp6 and *Cupriavidus* sp. IDO have comparatively high ANI values when compared with *C*. *necator* A5-1 and *R*. *pickettii* DTP0602 (Table S4), this clustering is appropriate. However, *C*. *oxalaticus* NBRC 13593, which had an ANI score of >90% when compared with *Cupriavidus* sp. amp6, was noticeably separated from cluster G and was instead categorized into cluster B, comprised of *C*. *taiwanensis, C*. *nantongensis*, and *C*. *alkaliphilus* strains. Cluster K contained several species, including *C*. *pauculus* KF709, *C*. *pinatubonensis* JMP134, *Cupriavidus* sp. BIS7, *Cupriavidus* sp. YR651, and *Ralstonia* sp. PBA. Surprisingly, the genome features of *Ralstonia* sp. PBA were more similar to those of *Cupriavidus* strains than to *Ralstonia* strains. As expected, *R*. *pickettii* DTP0602 and *Ralstonia* sp. 25fmcol4.1 were categorized into the *Cupriavidus*-derived clusters (Table 3). *Ralstonia* sp. A12 was classified into cluster N, consisting mainly of *R*. *insidiosa* strains. This classification is inconsistent with the results of MLSA, but similar to the results of ANI analysis. *R*. *solanacearum* strains (cluster P) were not separated into three groups as shown in the ANI matrix, but instead formed a single grouping. Cluster designations of all other *Cupriavidus* and *Ralstonia* strains based on TNA were the same as those determined by ANI analysis.

ANI analysis and TNA produced contradictory results in the designation of the several strains (*C*. *oxalaticus* NBRC 13593, *C*. *pauculus* KF709, *C*. *pinatubonensis* JMP134, *Cupriavidus* sp. BIS7, *Cupriavidus* sp. YR651, and *Ralstonia* sp. PBA) (Table 3). Richter and Rossello-Mora (Richter and Rossello-Mora, 2009) considered two scenarios as the causes for this paradoxical observation; (i) evolutionary or environmental forces may impede modifications of the genomic signature, resulting in a tetra-nucleotide frequency that does not reflect the actual phylogenetic position of the strain, and (ii) the amount of aligned sequence should be taken into account when ANI analysis was performed. We confirmed that adequate amounts of sequence were used for pairwise DNA sequence alignment in our analysis. When compared with the results of phylogenetic analyses and ANI analysis, we propose that ANI method is more reliable and suitable for inferring phylogenetic relationships as the results are more clear cut than TNA method.

### Reclassification of the Strains of the Genera *Cupriavidus* and *Ralstonia*

The proposed reclassification of several *Cupriavidus* and *Ralstonia* strains is summarized in Table 4. Phylogenetic and whole-genome sequence analyses confirmed that strain NH9 belongs to the species *C*. *necator*, but also suggested that 41 of the strains examined in this study should be corrected in terms of their taxonomic classifications. While phylogenetic analysis did not support a change in taxonomic classification for *C*. *basilensis* KF708 and *C*. *necator* A5-1, whole-genome sequence analyses suggested that the species designations of these strains were incorrect. The classification of *R*. *pickettii* DTP0602 was also called into question based on the results of whole-genome sequence analyses (Table 4). Overall, these results suggest that the combination of phylogenetic and whole-genome sequence analyses can identify the correct taxonomic assignments for bacterial strains, with whole-genome sequence analyses being particularly useful for improving the resolution, although biochemical characterization is required for complete taxonomic classification.

**Table 4:** Summary of proposed reclassification of *Cupriavidus* and *Ralstonia* strains.

**GenBank strain name**	**Classification**		**Phylogenetic analyses**		**Whole-genome sequence analyses**		
	**Current**	**Proposed**	**16S**	**MLSA**	**ANI**	**TNA**	**POCP**
KF708	*C*. *basilensis*	*Cupriavidus* sp.	*C*. *basilensis*	*C*. *basilensis*	+	+	*Cupriavidus*
A5-1	*C*. *necator*	*Cupriavidus* sp.	*C*. *necator*	*C*. *necator*	+	+	*Cupriavidus*
NH9	*C*. *necator*	*C*. *necator*	+	+	+	+	*Cupriavidus*
UM1	*C*. *pauculus*	*Cupriavidus* sp.	+	+	+	+	*Cupriavidus*
GA3-3	*Cupriavidus* sp.	*C*. *necator*	N. T.	+	+	+	*Cupriavidus*
HMR-1	*Cupriavidus* sp.	*C*. *metallidurans*	+	+	+	+	*Cupriavidus*
HPC(L)	*Cupriavidus* sp.	*C*. *gilardii*	+	+	+	+	*Cupriavidus*
SHE	*Cupriavidus* sp.	*C*. *metallidurans*	+	+	+	+	*Cupriavidus*
SK-4	*Cupriavidus* sp.	*C*. *necator*	+	+	+	+	*Cupriavidus*
UYPR2.512	*Cupriavidus* sp.	*C*. *necator*	N. T.	+	+	+	*Cupriavidus*
DTP0602	*R*. *pickettii*	*Cupriavidus* sp.	*C*. *necator*	*C*. *necator*	+	+	*Cupriavidus*
FDAARGOS_410	*R*. *pickettii*	*R*. *mannitolilytica*	+	+	+	+	*Ralstonia*
CQPS-1	*R*. *solanacearum*	*R*. *pseudosolanacearum*	+	+	+	*R*. *solanacearum*	*Ralstonia*
EP1	*R*. *solanacearum*	*R*. *pseudosolanacearum*	+	+	+	*R*. *solanacearum*	*Ralstonia*
P781	*R*. *solanacearum*	*R*. *pseudosolanacearum*	+	+	+	*R*. *solanacearum*	*Ralstonia*
UW757	*R*. *solanacearum*	*R*. *pseudosolanacearum*	+	+	+	*R*. *solanacearum*	*Ralstonia*
CaRs-Mep	*R*. *solanacearum*	*R*. *pseudosolanacearum*	+	+	+	*R*. *solanacearum*	*Ralstonia*
CFBP3059	*R*. *solanacearum*	*R*. *pseudosolanacearum*	+	+	+	*R*. *solanacearum*	*Ralstonia*
KACC10709	*R*. *solanacearum*	*R*. *pseudosolanacearum*	+	+	+	*R*. *solanacearum*	*Ralstonia*
KACC 10722	*R*. *solanacearum*	*R*. *syzygii*	+	+	+	*R*. *solanacearum*	*Ralstonia*
OE1-1	*R*. *solanacearum*	*R*. *pseudosolanacearum*	+	+	+	*R*. *solanacearum*	*Ralstonia*
RSCM	*R*. *solanacearum*	*R*. *pseudosolanacearum*	+	+	+	*R*. *solanacearum*	*Ralstonia*
SEPPX05	*R*. *solanacearum*	*R*. *pseudosolanacearum*	+	+	+	*R*. *solanacearum*	*Ralstonia*
YC40-M	*R*. *solanacearum*	*R*. *pseudosolanacearum*	+	+	+	*R*. *solanacearum*	*Ralstonia*
58_RSOL	*R*. *solanacearum*	*R*. *insidiosa*	+	+	+	+	*Ralstonia*
PSS4	*R*. *solanacearum*	*R*. *pseudosolanacearum*	+	+	+	*R*. *solanacearum*	*Ralstonia*
PSS190	*R*. *solanacearum*	*R*. *pseudosolanacearum*	+	+	+	*R*. *solanacearum*	*Ralstonia*
PSS216	*R*. *solanacearum*	*R*. *pseudosolanacearum*	+	+	+	*R*. *solanacearum*	*Ralstonia*
PSS1308	*R*. *solanacearum*	*R*. *pseudosolanacearum*	+	+	+	*R*. *solanacearum*	*Ralstonia*
RD15	*R*. *solanacearum*	*R*. *pseudosolanacearum*	+	+	+	*R*. *solanacearum*	*Ralstonia*
Rs-T02	*R*. *solanacearum*	*R*. *pseudosolanacearum*	+	+	+	*R*. *solanacearum*	*Ralstonia*
UTT-25	*R*. *solanacearum*	*R*. *pseudosolanacearum*	+	+	+	*R*. *solanacearum*	*Ralstonia*
25mfcol4.1	*Ralstonia* sp.	*Cupriavidus* sp.	+	+	+	+	*Cupriavidus*
5_2_56FAA	*Ralstonia* sp.	*R*. *pickettii*	+	+	+	+	*Ralstonia*
A12	*Ralstonia* sp.	*R*. *insidiosa*	+	*R*. *pickettii*	*Ralstonia* sp.	+	*Ralstonia*
AU12-08	*Ralstonia* sp.	*R*. *insidiosa*	+	+	+	+	*Ralstonia*
MD27	*Ralstonia* sp.	*R*. *insidiosa*	+	+	*Ralstonia* sp.	+	*Ralstonia*
NFACC01	*Ralstonia* sp.	*R*. *pickettii*	+	+	+	+	*Ralstonia*
NT80	*Ralstonia* sp.	*R*. *insidiosa*	+	+	+	+	*Ralstonia*
UNC404CL21Col	*Ralstonia* sp.	*R*. *pickettii*	+	+	+	+	*Ralstonia*
UNCCL144	*Ralstonia* sp.	*R*. *pickettii*	+	+	+	+	*Ralstonia*
PBA	*Ralstonia* sp.	Other	+	*Cupriavidus* sp.	+	*Cupriavidus* sp.	+

*The symbol “+” indicates same taxonomy as “Proposed.” N. T., Not tested*.

All analysis methods clearly indicated that *R*. *pickettii* DTP0602 and *Ralstonia* sp. 25mfcol4.1 should be reclassified into the genus *Cupriavidus*. Strain DTP0602 has also previously been flagged for reclassification into the genus *Cupriavidus* (Zhang and Qiu, 2016), but robust taxonomic analysis has not been performed until now. In contrast, the current study is the first to reveal strain 25mfcol4.1 as a member of the genus *Cupriavidus*. Interestingly, three different species, *C*. *alkaliphilus, C*. *nanotongensis*, and *C*. *taiwanensis* showed high similarities in all analyses. ANI-based categorization (cut-off value 90%), especially, suggested that these bacteria are the same species (ANI values > 92%), although the three species have been already reported to be separated via phenotypic characterization and DNA-DNA hybridization (Sun et al., 2016). Strains H16 and JMP134 (formerly known as *R. eutropha*) were reported to share high similarities with *C*. *necator* and *C*. *pinatubonensis* type-strains, respectively, based on DNA-DNA hybridization analyses (Vandamme and Coenye, 2004; Sato et al., 2006). However, detailed taxonomic experiments, and therefore a robust classification, have never been performed for strain H16. Our results of 16S rRNA gene-based phylogenetic analysis, MLSA, ANI, and TNA agreed with previous classifications. By using several discrimination methods, the taxonomic positions of strains H16 and JMP134, which have been widely studied as a polyhydroxybutyrate producer (Pohlmann et al., 2006; Kutralam-Muniasamy and Perez-Guevara, 2018) and a 2,4-dichlorophenoxyacetic acid degrader (Lykidis et al., 2010), respectively, have been clarified in the current study.

The species *R*. *solanacearum* were clearly separated into three subgroups based on phylogenetic and ANI analyses, and this result was consistent with previous studies (Safni et al., 2014; Prior et al., 2016; Zhang and Qiu, 2016). In our research, newly 20 strains were proposed to be reclassified into appropriate taxonomic positions (Table 4). Contradictory results were obtained regarding the phylogenetic position of *Ralstonia* sp. A12, as described above. Considering all of the results for this strain, we concluded that it could also be classified as *R*. *insidiosa* (Table 4). Although a species classification could not be determined for *Ralstonia* sp. MD27 at an ANI value of 95%, phylogenetic analyses and TNA clearly indicated that it belongs to the species *R*. *insidiosa*. While *Ralstonia* sp. PBA shows phylogenetic affinity to the genus *Cupriavidus* based on 16S rRNA nucleotide sequence analysis (Figure 4) and tetra-nucleotide usage (Figure 9), it is unlikely to belong to either the genus *Cupriavidus* or the genus *Ralstonia* based on POCP identities of <60% when compared with *Cupriavidus* and *Ralstonia* strains (Figure 8). Based on POCP analysis, Kim and Gan proposed that *Ralstonia* sp. PBA should be classified into the genus *Cupriavidus*; however, they only performed pairwise comparison of the proteome of strain PBA with proteins from *Cupriavidus* and *Burkholderia* strains (Kim and Gan, 2017). Regardless, all results presented so far confirm that strain PBA is a member of the family *Burkholderiaceae*. A more comprehensive analysis including strains belonging to related genera will provide a more appropriate classification of strain PBA.

## Conclusion

In the present work, the complete genome sequence of *C*. *necator* NH9 was obtained. Analyses of general genome properties, genome structure, and the aromatic compound degradation capacity of NH9 demonstrated that this bacterium had similar characteristics to other *Cupriavidus* strains. The presence of several dioxygenase-encoding genes suggested a versatile role for NH9 in the degradation of aromatic compounds in contaminated soil. Based on comprehensive phylogenetic and genomic analyses, NH9 was clearly identified as belonging to the species *C*. *necator*. Further analyses of 46 *Cupriavidus* and 104 *Ralstonia* strains also indicated that 41 of these strains should be reclassified at either the genus or species level. In particular, two *Ralstonia* strains should be reclassified into the genus *Cupriavidus*. The combination of several discrimination methods allowed more precise classification of these bacteria, which have a complex taxonomic history. We determined that the ANI method was a particularly powerful tool for classification of bacteria at the species level. However, we propose that standard ANI cut-off values of 90 and 95% be applied to *Cupriavidus* and *Ralstonia* strains, respectively, because the species diversity within the genus *Cupriavidus* is higher than that of the genus *Ralstonia*. In addition, a 90% ANI threshold should also be applied to the species *R*. *pickettii* because of its similarly high level of diversity. On the other hand, while the phylogenetic re-location of the strain DTP0602 as a species of *Cupriavidus* by our analysis turned out to accord with the tendency of degradation ability of aromatic compounds by *Cupriavidus*, incongruence was observed between the delineation of the two genera and the tendency of the aromatic degradation abilities (Figure S2). This suggested that some genetic events such as horizontal transfer of the degradation genes in the past beyond the genus.

## Author Contributions

RM, HD, and NO conceived and designed the experiments. RM performed the experiments, analyzed the data, prepared all tables and figures, and wrote the manuscript. HD and YK provided assistance with analysis tools. HD, YK, and NO critically reviewed and curated the manuscript. NO is responsible for the project.

### Conflict of Interest Statement

The authors declare that the research was conducted in the absence of any commercial or financial relationships that could be construed as a potential conflict of interest.

## Abbreviations

3-CB: 3-chlorobenzoate
ANI: average nucleotide identity
CDSs: coding sequences
Chr: chromosome
COG: clusters of orthologous groups
Inc: incompatibility
KEGG: kyoto encyclopedia of genes and genomes
MiGAP: microbial genome annotation pipeline
MLSA: multilocus sequence analysis
PAHs: polycyclic aromatic hydrocarbons
PCA: principle component analysis
PCBs: polychlorinated biphenyls
PGAAP: prokaryotic genome automatic annotation pipeline
POCP: percentage of conserved proteins
TNA: tetra-nucleotide analysis.
