# Supplementary material for: Complete Genome Sequence of 3-Chlorobenzoate-Degrading Bacterium Cupriavidus necator NH9 and Reclassification of the Strains of the Genera Cupriavidus and Ralstonia Based on Phylogenetic and Whole-Genome Sequence Analyses
Source: Front Microbiol. 2019 Feb 12;10:133. doi: 10.3389/fmicb.2019.00133 (PMC6379261; doi:10.3389/fmicb.2019.00133)
Supplement: Supplementary file 5 [file Data_Sheet_1.PDF]

Supplementary Material

Complete genome sequence of 3-chlorobenzoate-degrading bacterium *Cupriavidus necator* NH9 and reclassification of the strains of the genera *Cupriavidus* and *Ralstonia* based on phylogenetic and whole-genome sequence analyses

Ryota Moriuchi[1,2], Hideo Dohra[1], Yu Kanesaki[1] and Naoto Ogawa[2,3*]

[1]Research Institute of Green Science and Technology, Shizuoka University, 836 Ohya, Suruga-ku, Shizuoka-shi, Shizuoka 422-8529, Japan

[2]The United Graduate School of Agricultural Science, Gifu University, 1-1 Yanagido, Gifu-shi, Gifu 501-1193, Japan

[3]Graduate School of Agriculture, Shizuoka University, 836 Ohya, Suruga-ku, Shizuoka-shi, Shizuoka 422-8529, Japan

Correspondence:

Naoto Ogawa

Graduate School of Agriculture, Shizuoka University,

836 Ohya, Suruga-ku, Shizuoka-shi, Shizuoka, 422-8529, Japan.

E-mail address: ogawa.naoto@shizuoka.ac.jp

Tel/Fax: +81-54-238-4875/+81-54-237-3028

The Supplementary Material includes:

- Supplementary Materials and Methods
- Figures S1 and S2
- Tables S1-S4 (provided in Excel format)
- Reference

Supplementary Materials and Methods

Orthologous gene search

Orthologous genes which are supposed to be involved in degradation of 3-chlorocatechol, benzoate, phenol, 2-, 3-, or 4-hydroxybenzoate, 2,3-dihydroxybenzoate, benzoylformate, benzonitrile, catechol and 3-oxoadipate were annotated using the Kyoto Encyclopedia of Genes and Genomes (KEGG) database. This is because each sequence in the KEGG database has an associated KEGG Orthology term, which is in turn coupled to proteins whose functions have been experimentally verified (Kanehisa et al., 2016) (Supplementary Table S2). Genes not assigned in the KEGG database were searched and identified using BLASTP analysis. Strains registered in the KEGG database were used for search of orthologous genes and listed below: *C. basilensis* 4G11, *C. gilardii* CR3, *C. metallidurans* CH34, *C. necator* H16, *C. necator* N-1, *C. pinatubonensis* JMP134, *C. taiwanensis* LMG19424, *Cupriavidus* sp. USMAHM13, *Cupriavidus* sp. USMAA1020, *Cupriavidus* sp. USMAA2-4, *R. insidiosa* FC1138, *R. mannitolilytica* SN82F48, *R. pickettii* 12D, *R. pickettii* 12J, *R. pickettii* DTP0602 (which we propose should be renamed *Cupriavidus* sp.), *R. solanacearum* GMI1000, *R. solanacearum* FQY_4, *R. solanacearum* Po82, *R. solanacearum* CMR15, *R. solanacearum* PSI07, *R. solanacearum* CFBP2957, and *R. pseudosolanacearum* RS 476. Strains CFBP2957 and RS 476 are not used for reclassification in this study.

Prediction of potential abilities to degrade aromatic compounds

Based on the results of orthologous gene search and KEGG pathway analysis, aromatic compounds degradation capacities in selected *Cupriavidus* and *Ralstonia* strains were predicted and depicted as heatmap (Supplementary Figure S2). A dendrogram was constructed based on the complete clustering method in R program version 3.4.4 (https://www.r-project.org/).

Reference

Kanehisa, M., Sato, Y., Kawashima, M., Furumichi, M., and Tanabe, M. (2016). KEGG as a reference resource for gene and protein annotation. *Nucleic Acids Res* 44(D1), D457-462. doi: 10.1093/nar/gkv1070.

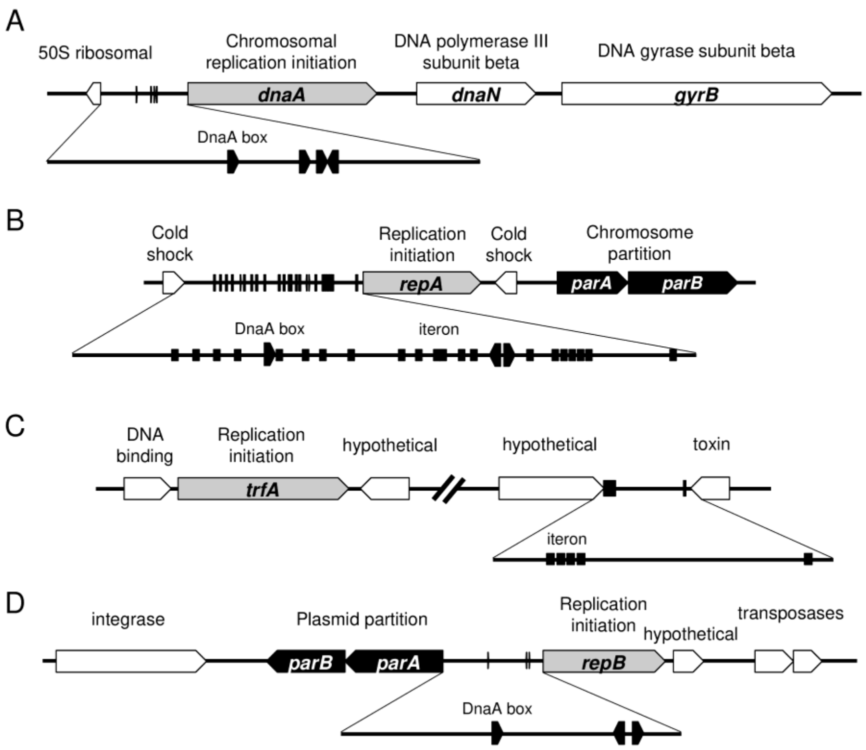

Figure S1. Schematic diagram of the regions containing putative replication origin and genes encoding replication initiation protein on four replicons of NH9. A. chromosome 1; B. chromosome 2; C. pENH91; and D. pENH92. Putative replication initiation genes, partitioning genes and other genes are colored in gray, black and white respectively. DnaA boxes and iteron sequences are shown by pentagon and square respectively.

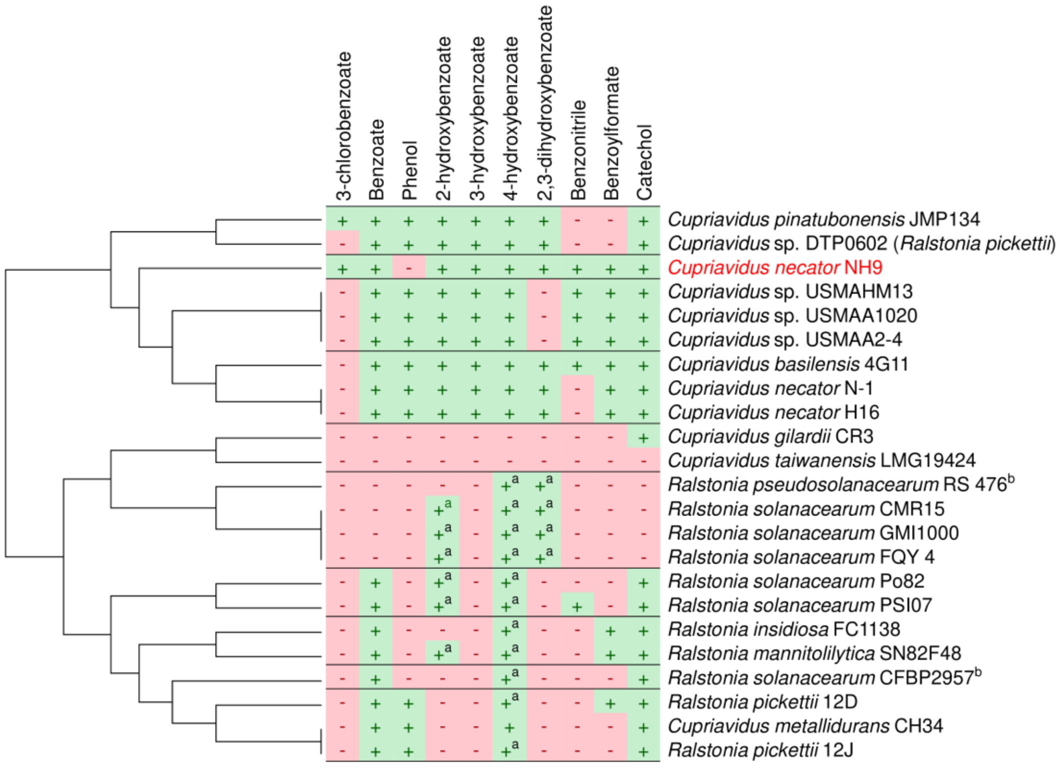

Figure S2. Heatmap and dendrogram of potential abilities to degrade aromatic compounds predicted by genomic features of *Cupriavidus* and *Ralstonia* strains. The symbols "+" and "-" indicate potential abilities and no potential abilities to degrade individual compound, respectively. Degradation capacities predicted by genes which are not described in Supplementary Table S2 were designated with a superscript "a". Strains not used for reclassification in this study were designated with a superscript "b". Strain DTP0602 is proposed to be reclassified into the genus *Cupriavidus* in this study. *Cupriavidus necator* NH9 was noted in red.
